# KSEMAW: an open source software for the analysis ofspectrophotometric, ellipsometric andphotothermal deflection spectroscopy measurements

**DOI:** 10.12688/openreseurope.13842.1

**Published:** 2021-08-18

**Authors:** Marco Montecchi, Alberto Mittiga, Claudia Malerba, Francesca Menchini

**Affiliations:** 1 Energy Technologies and Renewable Sources Department, ENEA Research Center Casaccia, Roma, 00123, Italy; 2 Department of Information Engineering, Electronics and telecommunications, University of Roma, Roma, Italy

**Keywords:** thin film, optical characterization, complex refractive index, film thickness, spectrophotometric measurements, ellipsometric measurements, open source software

## Abstract

The optical behavior of devices based on thin films is determined by complex refractive index and thickness of each slab composing the stack; these important parameters are usually evaluated from photometric and/or ellipsometric spectral measurements, given a model of the stack, by means of dedicated software. In the case of complex multilayer devices, generally a number of simpler specimens (like single-film on substrate) must be preliminarily characterized. This paper introduces the reader to a new open source software for thin film characterization finally released after about 30 years of development. The software has already been used in various fields of physics, such as thin film optical filters, architectural glazing, detectors for high energy physics, solar energy, and, last but not least, photovoltaic devices. Code source files, user manual as well as a sample of working directories populated with assorted files can be freely downloaded from the kSEMAW GitHub repository.

## Plain language summary

kSEMAW software is a useful tool for scientists and technicians dealing with optical devices based on optical coatings. More precisely, kSEMAW is a workspace for the analysis of Spectrophotometric (SP), Ellipsometric (ELI) and Photothermal Deflection Spectroscopy (PDS) measurements. The letter “k” indicates the use of the Qt libraries. Main features: (1) simulate SP, ELI and PDS measurements of a multilayer structure, being known the thicknesses and the complex refractive indexes of each material composing the different layers, (2)calculate the complex refractive index and the thickness of a given layer (if “thin” ) from experimental measurements (SP, ELI, PDS), being known the thicknesses and the complex refractive indexes of all the other layers composing the structure, (3)evaluate the mean value of physical quantities, weighted on a given international standard spectrum (such as ASTM G173-03) or on own customized reference spectrum, and (4) predict the angular trend by using a realistic model or the equivalent model algorithm.

## Introduction

A huge variety of materials can be deposited as films on substrates by means of several well-established techniques, ranging from physical vapour deposition to the simple and economic sol-gel dipping ^1^. When the thickness of the film is comparable with the wavelength of an electromagnetic wave hitting the film surface, the transmittance (T) and reflectance (R) of the system are greatly affected by the interference occurring among the contributes originating from the air-film and film-substrate interfaces ^2^. This allows, to some extent, regulating transmittance, reflectance and absorptance through an appropriate choice of the type of material composing the film and the film thickness.

As an example the simplest antireflection (AR) coating at the wavelength *λ* for a transparent window (lens) with refractive index *n* *_sub_* is obtained by depositing a material with refractive index $n≃\sqrt{n_{sub}},$ and thickness *d* satisfying the condition *nd* ≃ *λ/*4; for that reason it is called *quarter wavelength anti-reflection coating*.

In order to shape transmittance and reflectance of the device to an arbitrary structured spectrum, one has to adopt a suitable multi-layer coating, i.e. a stack of several films, where the sequence of different materials and their thicknesses are optimized to get the desired goal.

Thin film technology has become so well established that today everyone, in everyday life, uses many devices based on optical coatings, mono, and multi-layers ^1^.

Whenever a new material has to be introduced in a coating, its optical characterization is needed to optimize the growth process: as a matter of fact, every deposition set-up is different from another, making the accurate tuning of the many deposition parameters, like vacuum (if needed), temperature, growth rate, etc, mandatory in order to obtain a film sufficiently adherent to the substrate, with the proper refractive index, that is homogeneous all over its surface, is resistant to cleaning, and so on.

Such an optimization process runs by depositing a number of films on one or more substrate types; each specimen has to be optically characterized by measuring its relevant optical features (like *T* and *R* at normal or oblique incidence, or the ellipsometric angles Δ and Ψ) from which the complex refractive index of the material and the film thickness are evaluated.

To this aim the software herein presented, under development since 1988, sets an environment where the user can:

• load experimental spectra of different kinds, like photometric, ellipsometric and absorbance detected with a photothermal deflection spectroscopy apparatus (PDS)
• set up an optical model of the sample
• calculate the refractive index and the thickness of the film.

The software is named kSEMAW (Spectro-Ellipsometric Measurement Analysis Workbench), where the letter “k” indicates the use of the Qt libraries to generate the graphical user interface, typical of the Linux KDE desktop environment. Over the years kSEMAW has been expanded to meet the ever-changing needs encountered along the career-path of the corresponding-author, such as:

• optical characterization of thin films ^3^
• evaluation of luminous and energetic features of architectural glazing ^4^
• optical characterization of scintillator crystal ( *PbWO*_4_) ^5^, glue ^6^ and detector (APD) ^7^ used in the CMS experiment ^8^ at LHC of CERN
• optical characterization of mirrors ^9^ and anti-reflection coatings used in concentrated solar power (CSP)
• last, but not least, optical characterization of tandem photovoltaic cells.

This explains the considerable development that has taken place over the years, accompanied by the replacement of command line with a modern graphic user interface (GUI); this has greatly increased the software ease of use. As a matter of fact, although kSEMAW was born for personal use, like a sort of Swiss knife, the last improvements make it a very interesting tool for anyone working in thin film technology.

To add value to the work done so far, believing in knowledge sharing, we took the opportunity offered by Open Research Europe to make kSEMAW available for everyone as open source software under the GNU General Public License as published by the Free Software Foundation version 3. Code source files, user manual as well as a sample of working directories populated with assorted files can be freely downloaded from here.

In this article, after a brief presentation of kSEMAW, rather than replicating the user manual, we will show the main capabilities of the software through concrete examples; all the related files are part of the example working directories, so that interested users can replicate the computing for any example here shown on their own software installation.

## What kSEMAW can do

kSEMAW is a workspace for the analysis of Spectrophotometric (SP), Ellipsometric (ELI) and Photothermal Deflection Spectroscopy (PDS) measurements.

kSEMAW allows to:

1. simulate SP, ELI and PDS measurements of a multilayer structure, knowing the thicknesses and the complex refractive indexes $\tilde{n}$ = *n* – i *k* of each material composing the different layers ^1^;
2. calculate the complex refractive index *n* – i *k* and the thickness of a given layer (if “thin”) from experimental measurements (SP, ELI, PDS), knowing the thicknesses and the complex refractive indexes of all the other layers composing the structure;
3. evaluate the mean value of physical quantities (for example transmittance or reflectance), weighted on a given international standard spectrum (such as the illuminant D65 or the direct solar spectrum ASTM G173-03) or on a customized reference spectrum (for example the crystal scintillator PbWO _4_ luminescence spectrum, used in the CMS experiment at CERN LHC);
4. predict reflectance/transmittance angular trend, once the realistic model of the coating has been set or by means of the equivalent model algorithm ^10^; where necessary output spectra can be weighted over a given reference spectrum.

kSEMAW uses a mathematical approach based on transfer matrices, particularly suitable for the case of multilayers: in addition to being able to treat coherent propagation and interference (for arbitrary incidence angles), it also allows to simulate the effects due to different types of non-ideal material properties, such as optical constants gradients along the film thickness, thickness inhomogeneity, porosity and roughness. The latter is modelled by assuming the thickness to be Gaussian-like distributed with standard deviation *σ*, and variations along distances much greater than the wavelength *λ*. The program deals with moderately rough interfaces ( *σ* ≪ *λ*) and limited radiation diffusion ^11^.

kSEMAW can model devices composed by up to 9 layers of different materials. For each layer it is possible to: i) consider it as optically *thin* or *thick* with respect to the radiation coherence length ^2^, for summing up coherently or not the contributes originating from its two interfaces in wave propagation; ii) set a refractive index profile along the thickness; iii) introduce roughness at the outermost interface; iv) consider the layer as composed of a mixture of two different materials with optical constants calculated on the basis of the Effective Medium Approximation method. The latter feature can be used to model porosity, by considering the layer as composed by a mixture of material and voids (air) in an given percentage.

## Methods

To obtain the unknown *n* and *k* values of a film starting from experimental measurements, kSEMAW uses two different methods:

• solution-tracking search method;
• hybrid computational method, called “IbridOne”.

### Solution-tracking search method

The first step of the solution-tracking method is to search for a ( *n*, *k*) couple of values that reproduces the experimental measurements (for example *T* & *R* or Δ & Ψ) at a given *λ* (typically *λ* *_min_* and *λ* *_max_* of the considered wavelength range). The search is separately performed for each experimental measurement within the region of the ( *n*, *k*) space chosen by the user: the *n*-interval is sampled with 51 points and, for each one of them, the *k* value which reproduces the experimental data is found by means of a bisection method; then a similar procedure is repeated by exchanging *n* with *k*. In this way, for each measurement a solution-belt is obtained in the ( *n*, *k*) space, limiting the ( *n _λ_*, *k _λ_*) values allowing reproducing experimental data; the belt width is determined by the experimental error. The different solution-belts corresponding to the two (or more) considered measurements are displayed in a dedicated window and their cross section represents the wanted common ( *n*, *k*)-solution (see use case #1 as example).

To ensure good accuracy it is necessary to choose at least a couple of measurements *M*_1_ & *M*_2 _(like *T* & *R* or Δ & Ψ) that give rise to solution-belts almost orthogonal one to the other. Sometimes multiple belt crossings are present. In any case the user is asked to select the more appropriate initial values at *λ* *_min_* and/or *λ* *_max_*.

Once the initial values have been set, the user can launch the tracking method: starting from the minimum or maximum wavelength the solutions *n*_λ _and *k*_λ _of the following wavelength are numerically computed by minimizing the merit function $MF=\sum_{i}{\left(M_{i,cal}(n,k)-M_{i,exp}\right)}^{2}/\text{Δ}M_{i}^{2}$ . The process is sequentially run over all the wavelengths sampling the wavelength range.

As the search progresses, the solutions *n*_λ _and *k*_λ _are plotted in two dedicated windows describing a portion of the spaces ( *λ*, *n*) and ( *λ*, *k*). At the end of the process, by observing the set of solutions in the ( *λ*, *n*) space, one can note that zero, one or more solutions can exist for each wavelength in the case of thin film; in addition, the set of solutions is not necessarily connected. By optimizing the model parameters (thicknesses, roughness, gradients, etc.) according to specific guiding criteria ^12, 13^, a more connected set of solutions can be obtained (see use case #2); finally, among them, a physically reasonable subset of solutions must be selected.

Beside the above described pure sequential search of solutions, when the material is expected to be quite similar to previously study specimens, the user can set a different strategies for solution search; referring to the user manual for more information, here we just recall the possibility to set the values of a given *nk*-file as initial values to start the search at each wavelength.

### IbridOne computational method

In the IbridOne method:

1. *n*( *λ*) is modelled with appropriate analytical functions;
2. the extinction coefficient *k*( *λ*) is numerically computed from *T*( *λ*) and the assumed *n*( *λ*);
3. the computed reflectance *R _comp_* ( *λ*) is compared with the experimental one by means of a merit function;
4. some parameters of the assumed *n*( *λ*) analytical function as well as of the optical model are optimized by a non-linear least square algorithm.

### Analytical functions for modelling *n*( *λ*)

Concerning the analytical functions, they may be in principle arbitrary but it is much more convenient that they possess a clear physical interpretation. Theories for the light-matter interaction give expressions for the dielectric susceptibility and the simplest ones are those representing the complex dielectric susceptibility $\tilde{\chi }$ = *χ*_1_ – i *χ*_2_ associated to single resonant oscillators, obtained from both classical (Drude–Lorentz oscillator) or quantum approach. The dielectric susceptibility is connected to the permittivity $\tilde{}$ by the equation


$$\;\tilde{}={}_{1}-\text{i}{}_{2}=1+\tilde{\chi }=1+\chi_{1}-\text{i}\chi_{2}\;(1)$$

In turn refractive index and permittivity are both complex quantities connected by the relationships


$$\;{}_{1}=n^{2}-k^{2},\;{}_{2}=2nk\;(2)$$

and inversely


$$\;n=\frac{1}{\sqrt{2}}\sqrt{\sqrt{{}_{1}^{2}+{}_{2}^{2}}+{}_{1}},\;k=\frac{1}{\sqrt{2}}\sqrt{\sqrt{{}_{1}^{2}+{}_{2}^{2}}-{}_{1}}\;(3)$$

In the current version, kSEMAW can model the dependence of the refractive index on wavelength *n*( *λ*) using up to 20 “oscillators” (i.e. analytical functions) belonging to the following 9 classes:

• Flat
• Lorentz
• Quant-homo
• Quant-inhomo
• Drude
• Direct Gap Cody
• Direct Gap Tauc
• Indirect Gap Cody
• Indirect Gap Tauc

kSEMAW calculates the total $\tilde{}$ adding up the susceptibilities of all the selected oscillators. The complex refractive index will be then calculated using Equation 3.

The oscillator functions are described in detail in Appendix A of the user manual available in the kSEMAW GitHub repository ^14^. Here we just give a brief description for each one.

The “Flat" oscillator is a simple real constant. One “Flat" oscillator must be always inserted to take into account the term “1” in Equation 1 (i.e. the vacuum permittivity *ϵ*_0_) as well as the tail-contribution of the transitions at high energies, out of the investigated wavelength range, and not explicitly considered.

The “Lorentz” oscillator is based on the classic Lorentz oscillator formula ^15^:


$$\;{\tilde{\chi }}_{lo}=C\left[\frac{\left(E_{r}^{2}-E^{2}\right)}{{\left(E_{r}^{2}-E^{2}\right)}^{2}+{\left(ED\right)}^{2}}-\text{i}\frac{ED}{{\left(E_{r}^{2}-E^{2}\right)}^{2}+{\left(ED\right)}^{2}}\right]\;(4)$$

where *C* is the oscillator amplitude, *E* *_r_* is the resonance energy and *D* is the line width.

The “Quant-homo” oscillator is based on the simple quantum oscillator formulas ^16^ valid for the case of homogeneous broadening and its shape is given by:


$$\;{\tilde{\chi }}_{qo}(E,E_{r})=C\left[\frac{(E_{r}-E)/D}{1+{\left[(E_{r}-E)/D\right]}^{2}}-\text{i}\frac{1}{1+{\left[(E_{r}-E)/D\right]}^{2}}\right]\;(5)$$

As reported in 17, the “Quant-homo” oscillator formula is obtained by neglecting a not-resonant term in the quantum treatment (otherwise a formula practically equivalent to the Lorentz oscillator would be obtained). This approximation is excellent near the resonance energy while a small difference appears far away from it. On the other hand the simple and elegant equations obtained with this approximation will be quite useful in the following.

When the absorbing centres in a material cannot be considered as identical replicas, the absorption-line broadening is inhomogeneous. For example absorbing centres may be slightly different in a crystal because of random presence of strain or proximity to other lattice defects and impurities that are randomly distributed.“Quant-inhomo” is therefore obtained by making the convolution between a homogeneous quantum oscillator and a Gaussian distribution centred in *E* *_r_* and with half width at half maximum (HWHM) equal to *D* ^18^; the line width of the homogeneous quantum oscillator is assumed to be much lower than the Gaussian one.

The “Drude” oscillator describes the response of a free electron gas and can be obtained by setting *E* *_r_* = 0 in the “Lorentz” oscillator:


$$\;{\tilde{\chi }}_{dr}=-\frac{E_{0}^{2}}{D^{2}+E^{2}}-\text{i}\frac{E_{0}^{2}D}{E(D^{2}+E^{2})}.\;(6)$$

In this case, *D* is related to the carrier scattering time *τ* by *D* = *ħ* */τ* while *E*_0_ is given by $E_{0}^{2}$ = ( *ħ* ^2^ *Nq* ^2^)/( m* *ϵ*_0_) where *N* is the carrier density per unit volume and m* is the carrier effective mass.

These single resonant oscillators are unsuitable to describe the optical constants of systems with a continuous distribution of density of states such as semiconductors; therefore the last four functions, specific for semiconductor materials, have been added and treated with a new approach.

As a matter of fact the semiconductors theories reported in textbooks ^19^ usually derive *ϵ*_2_ = *χ*_2_ only and exclusively at energies near the fundamental gap. Furthermore the calculation is made by hypothesizing that the excited states have infinite lifetime, i.e. that every transition between two states may take place only by absorbing photons with energy exactly equal to the difference of their energetic levels; the real part *ϵ*_1_ is not explicitly calculated, but it can be obtained by means of the Kramers-Kronig relations. Since the Kramers-Kronig integrals have to be numerically computed (except for particular cases) that approach is not so convenient for best-fit procedures.

In kSEMAW analytical expressions for both *χ*_1_ and *χ*_2_, satisfying the Kramers-Kronig relations and having a clear physical interpretation, are used. These expressions are obtained performing the convolution between a suitable $\chi_{2}^{inf}$ calculated for infinite lifetime and the normalized ( *C* = 1 */*( *πD*)) complex ${\tilde{\chi }}_{qo}$ describing the response of a quantum oscillator ( Equation 5):


$$\;\tilde{\chi }(E)=\int \chi_{2}^{inf}(E_{r}){\tilde{\chi }}_{qo}(E_{r}-E)dE_{r}\;(7)$$

The result of the convolution can be expressed as analytical functions in a few simple but interesting cases discussed below.

In general $\chi_{2}^{inf}$ can be expressed as the product of the joint density of states *ρ*( *E*) and a transition intensity *I*( *E*) ^20^: 


$$\;\chi_{2}^{inf}(E)=I(E)\rho (E)\;(8)$$

*I*( *E*) contains the squared modulus of the matrix element of the electron-photon interaction Hamiltonian between the initial and final states. In the dipole approximation the Hamiltonian is proportional to the position operator ( *r*) but it can be written in an equivalent way using the momentum operator ( *p*) also. A relation between the matrix elements of these two operators can be obtained using commutator relations:


$$\;{\left|\text{〈}p\text{〉}\right|}^{2}={\left|\text{〈}r\text{〉}\right|}^{2}{\left(\frac{mE}{\hbar }\right)}^{2}\;(9)$$

The energy dependence of *I*( *E*) cannot be easily predicted and therefore it is assumed to be a constant if |〈 *r*〉| ^2^ is supposed to be independent of energy while it is assumed to be proportional to 1 */E* ^2^ if |〈 *p*〉| ^2^ is supposed to be independent of energy. The constant |〈 *p*〉| ^2 ^assumption was used by Tauc *et al.* ^21^ in the interpretation of the optical absorption of amorphous semiconductors leading to the well known "Tauc plot" method to derive the optical gap. The constant |〈 *r*〉| ^2^ assumption was later on proposed by Cody ^22^ again for the amorphous semiconductor case. These two alternatives can be used for crystalline materials too. For example in crystalline GaAs the energy dependence of the optical absorption above the bandgap is better described by the constant position matrix element ^20^ rather than by the more commonly hypothesized constant momentum matrix element.

For a crystalline semiconductor with direct allowed transitions between two parabolic bands separated by an energy gap *E*_0_, *ρ*( *E*) can be written as ^19^:


$$\;\rho (E)=C_{r}{\left(E-E_{0}\right)}^{1/2}\;(10)$$

This monotonically increasing expression for *ρ*( *E*) is obviously valid only for energies slightly larger than the gap. For higher energy values *ρ*( *E*) can show a rather complex behavior but it has also a high energy limit determined by the extension of the valence and conduction bands. At the gap *E*_0_ and at this high energy limit ( *E*_3_) the *ρ*( *E*) has two "critical points" called *M*0 and *M*3 where it goes to zero following a square root behavior ^19^.

The simplest expression valid for every energy which has the correct square root behaviour near *E*_0_ and *E*_3_ is:


$$\;\rho (E)=C_{r}\sqrt{\left(E-E_{0})(E_{3}-E\right)}\;(11)$$

Using this expression and the "Cody’s approximation" the $\chi_{2}^{inf}$ also has the same form:


$$\;\chi_{2-dir-Cody}^{inf}(E)=C\sqrt{\left(E-E_{0})(E_{3}-E\right)}\;(12)$$

**Figure 1.**
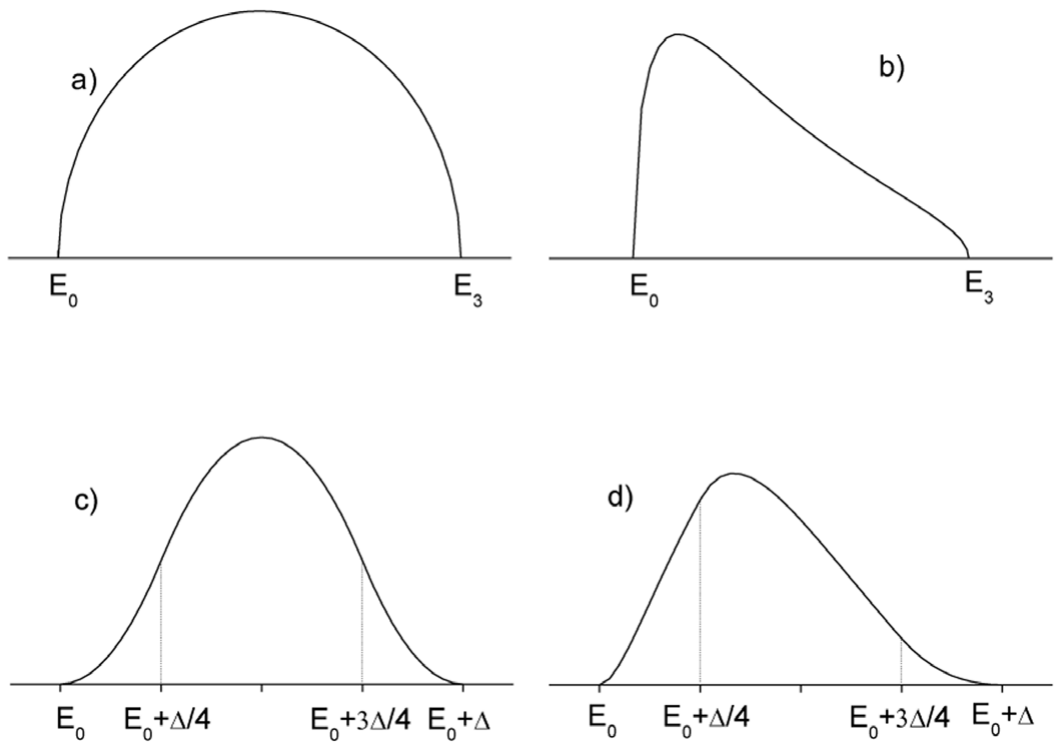
Imaginary part of the dielectric susceptibility for infinite lifetime semiconductor ( $\chi_{2}^{inf}$ ) and: **a**) direct gap under Cody’s approximation **b**) direct gap under Tauc’s approximation, **c**) indirect gap under Cody’s approximation **d**) indirect gap under Tauc’s approximation. *E*_0_ is the semiconductor gap. The high energy limit of the absorption band is labelled as *E*_3_ in **a**) and **b**) and as *E*_0_ + Δ in **c**) and **d**).

Equation 12 can be considered as an acceptable approximation for $\chi_{2}^{inf}$ ( *E*) in the case of a direct gap material with position matrix element independent of energy and with infinite lifetime (see Figure 1a).

On the other hand in Tauc’s approximation, the momentum matrix element is considered independent of energy and therefore, as previously discussed, the transition intensity is proportional to 1 */E* ^2^ (see Figure 1b), thus:


$$\;\chi_{2-dir-Tauc}^{inf}(E)=C\frac{\sqrt{\left(E-E_{0})(E_{3}-E\right)}}{E^{2}}\;(13)$$

Other types of critical points do exist ( *M*1 and *M*2) in the *ρ*( *E*) ^19^, but they are not yet included among the fitting functions of kSEMAW.

Both the complex permittivity components for these two cases can be obtained by the convolution between the functions in Equation 12 and Equation 13 and the normalized quantum oscillator ${\tilde{\chi }}_{qo}$ .

Direct gap Cody:


$$\begin{array}{l} \chi_{1-dir-Cody}(E)=\frac{CD}{2}\left\{a+b-2{\left(a^{2}+1\right)}^{1/4}{\left(b^{2}+1\right)}^{1/4}\cos\left[\frac{1}{2}\arctan\left(\frac{1}{a}\right)+\frac{1}{2}\arctan\left(\frac{1}{b}\right)\right]\right\}\\ \;=\frac{CD}{2}\left[a+b-2ℜ\left(\sqrt{a+i}\;\sqrt{b+i}\right)\right] \end{array}\;(14)$$

$$\begin{array}{l} \chi_{2-dir-Cody}(E)=-CD\left\{1+{\left(a^{2}+1\right)}^{1/4}{\left(b^{2}+1\right)}^{1/4}\sin\left[\frac{1}{2}\arctan\left(\frac{1}{a}\right)+\frac{1}{2}\arctan\left(\frac{1}{b}\right)\right]\right\}\\ \;=-CD\left[1-ℑ(\sqrt{a+i}\;\sqrt{b+i})\right] \end{array}\;(15)$$

where $a=\frac{E_{0}-E}{D}$ and $b=\frac{E_{3}-E}{D}$ .

Direct gap Tauc


$$\begin{array}{l} \chi_{1-dir-Tauc}(E)=\frac{C}{2D}\\ \;ℜ\{\frac{1}{2(D-iE)}\left(2iD+2E-2\sqrt{E_{0}E_{3}}-(i-1)\sqrt{2}\sqrt{D-i(E-E_{0})}\sqrt{-iD-E+E_{3}}\right)+\\ \;\frac{1}{2(D+iE)}\left(-2iD+2E-2\sqrt{E_{0}E_{3}}+(i+1)\sqrt{2}\sqrt{D+i(E-E_{0})}\sqrt{iD-E+E_{3}}\right)+\\ \;E{\left(D-iE\right)}^{2}\sqrt{D+i(E-E_{0})}\sqrt{D+i(E-E_{3})}/{\left(D^{2}+E^{2}\right)}^{2}+\\ \;E{\left(D+iE\right)}^{2}\sqrt{D-i(E-E_{0})}\sqrt{D-i(E-E_{3})}/{\left(D^{2}+E^{2}\right)}^{2}-\\ \;ED\left(D^{2}(E_{0}+E_{3})+E(-4E_{0}E_{3}+E(E_{0}+E_{3}))\right)/\left({\left(D^{2}+E^{2}\right)}^{2}\sqrt{E_{0}E_{3}}\right)\} \end{array}\;(16)$$

$$\begin{array}{l} \chi_{2-dir-Tauc}(E)=CD\\ \;ℜ\{-\left[{\left(D-iE\right)}^{2}\sqrt{D+i(E-E_{0})}\sqrt{D+i(E-E_{3})}\right]/\left(2D{\left(D^{2}+E^{2}\right)}^{2}\right)-\\ \;\left[{\left(D+iE\right)}^{2}\sqrt{D-i(E-E_{3})}\sqrt{D-i(E-E_{0})}\right]/\left(2D{\left(D^{2}+E^{2}\right)}^{2}\right)+\\ \;D\left[D^{2}(E_{0}+E_{3})+E(-4E_{0}E_{3}+E(E_{0}+E_{3}))\right]/\left(2D{\left(D^{2}+E^{2}\right)}^{2}\sqrt{E_{0}E_{3}}\right)\} \end{array}\;(17)$$

Different formulas must be used for materials where the fundamental gap is due to indirect transitions: in this case the excitation process includes the absorption or the emission of a phonon necessary to allow transitions between electronic states with different crystalline momentum. The absorption/emission of phonons removes the momentum conservation constraint and therefore for indirect transitions *ρ*( *E*) is proportional to the convolution of the density of states of the two bands. In amorphous semiconductors also the momentum conservation is not required because the disorder destroys translational simmetry and momentum is no more a good quantum number. It follows that oscillators for indirect transitions in crystalline semiconductors can be used to fit the optical constants of amorphous semiconductors too.

Analytical expressions for the convolution integral over the whole bands are not available, but it is well known that the convolution between two square root densities of states gives a function which increases parabolically above the gap. Therefore an empirical approximated expression can be obtained by joining two parabolic edges with a parabolic maximum, getting in Cody’s approximation (see Figure 1c):


$$\;\chi_{2-ind-Cody}^{inf}(E)=\;\frac{16C}{{\text{Δ}}^{\text{2}}}{\left(E-E_{0}\right)}^{2}\;\text{if}\;E_{0}<E<E_{0}+\text{Δ}/4\;(18)$$

$$\;\chi_{2-ind-Cody}^{inf}(E)=\;C\left\{2-\frac{16}{{\text{Δ}}^{\text{2}}}{\left[E-(E_{0}+\text{Δ}/2)\right]}^{2}\right\}\;\text{if}\;E_{0}+\frac{\text{Δ}}{4}<E<E_{0}+\frac{\text{3Δ}}{4}\;(19)$$

$$\;\chi_{2-ind-Cody}^{inf}(E)=\;\frac{16C}{{\text{Δ}}^{\text{2}}}{\left[E-(E_{0}+\text{Δ})\right]}^{2}\;\text{if}\;E_{0}+\frac{3\text{Δ}}{4}<E<E_{0}+\text{Δ}\;(20)$$

where *E*_0 _is the gap energy and Δ is the absorption band width.

In the Tauc’s approximation the above expressions must be divided by the term *E* ^2^. 

As for the direct gap case, both the complex permittivity components can be obtained by the convolution between these parabolic terms and the normalized quantum oscillator ${\tilde{\chi }}_{qo}$ .

Indirect gap-Cody


$$\begin{array}{l} \chi_{1-ind-Cody}(E)=C\{2IC_{Re}^{Cody}(E,E_{0}+3\text{Δ}/4)-2IC_{Re}^{Cody}(E,E_{0}+\text{Δ}/4)+\\ \;16/{\text{Δ}}^{2}[+I2_{Re}^{Cody}(E,E_{0},E_{0}+\text{Δ}/4)-I2_{Re}^{Cody}(E,E_{0},E_{0})\\ \;-I2_{Re}^{Cody}(E,E_{0}+\text{Δ}/2,E_{0}+3\text{Δ}/4)\;+I2_{Re}^{Cody}(E,E_{0}+\text{Δ}/2,E_{0}+\text{Δ}/4)\\ \;+I2_{Re}^{Cody}(E,E_{0}+\text{Δ},E_{0}+\text{Δ})-I2_{Re}^{Cody}(E,E_{0}+\text{Δ},E_{0}+3\text{Δ}/4)]\} \end{array}\;(21)$$

where:


$$\;IC_{Re}^{Cody}(E,E_{r})=\frac{1}{\pi D}\int \frac{[(E_{r}-E)/D]\text{d}E_{r}1+{\left[(E_{r}-E)/D\right]}^{2}}{=}\frac{1}{2\pi }\ln\left[D^{2}+{\left(E-E_{r}\right)}^{2}\right]\;(22)$$

$$\begin{array}{l} I2_{Re}^{Cody}(E,E_{0},E_{r})=\frac{1}{\pi D}\int \frac{{\left(E_{r}-E_{0}\right)}^{2}[(E_{r}-E)/D]\text{d}E_{r}}{1+{\left[(E_{r}-E)/D\right]}^{2}}=\\ \;\frac{1}{2\pi }\{(E_{r}-E)(3E-4E_{0}+E_{r})+4D(E-E_{0})\arctan\left(\frac{E-E_{r}}{D}\right)\\ \;+[{\left(E-E_{0}\right)}^{2}-D^{2}]\ln\left[D^{2}+{\left(E-E_{r}\right)}^{2}\right]\} \end{array}\;(23)$$

$$\begin{array}{l} \chi_{2-ind-Cody}(E)=C\{2IC_{Im}^{Cody}(E,E_{0}+3\text{Δ}/4)-2IC_{Im}^{Cody}(E,E_{0}+\text{Δ}/4)+\\ \;16/{\text{Δ}}^{2}[+I2_{Im}^{Cody}(E,E_{0},E_{0}+\text{Δ}/4)-I2_{Im}^{Cody}(E,E_{0},E_{0})\\ \;-I2_{Im}^{Cody}(E,E_{0}+\text{Δ}/2,E_{0}+3\text{Δ}/4)+I2_{Im}^{Cody}(E,E_{0}+\text{Δ}/2,E_{0}+\text{Δ}/4)\\ \;+I2_{Im}^{Cody}(E,E_{0}+\text{Δ},E_{0}+\text{Δ})-I2_{Im}^{Cody}(E,E_{0}+\text{Δ},E_{0}+3\text{Δ}/4)]\} \end{array}\;(24)$$

Where:


$$\;IC_{Im}^{Cody}(E,E_{r})=\frac{1}{\pi D}\int \frac{\text{d}E_{r}}{1+{\left[(E_{r}-E)/D\right]}^{2}}=-\frac{1}{\pi }\arctan\left(\frac{E-E_{r}}{D}\right)\;(25)$$

$$\begin{array}{l} I2_{Im}^{Cody}(E,E_{0},E_{r})=\frac{1}{\pi D}\int \frac{{\left(E_{r}-E_{0}\right)}^{2}\text{d}E_{r}}{1+{\left[(E_{r}-E)/D\right]}^{2}}=\frac{1}{\pi }(\left[D^{2}+{\left(E-E_{0}\right)}^{2}\right]\arctan\left(\frac{E-E_{r}}{D}\right)\\ \;+D\left\{E_{r}-E+(E-E_{0})\ln\left[D^{2}+{\left(E-E_{r}\right)}^{2}\right]\right\}) \end{array}\;(26)$$

Indirect gap-Tauc


$$\begin{array}{l} \chi_{1-ind-Tauc}(E)=C\{2IC_{Re}^{Tauc}(E,E_{0}+3\text{Δ}/4)-2IC_{Re}^{Tauc}(E,E_{0}+\text{Δ}/4)+\\ \;16/{\text{Δ}}^{2}[+I2_{Re}^{Tauc}(E,E_{0},E_{0}+\text{Δ}/4)-I2_{Re}^{Tauc}(E,E_{0},E_{0})\\ \;-I2_{Re}^{Tauc}(E,E_{0}+\text{Δ}/2,E_{0}+3\text{Δ}/4)+I2_{Re}^{Tauc}(E,E_{0}+\text{Δ}/2,E_{0}+\text{Δ}/4)\\ \;+I2_{Re}^{Tauc}(E,E_{0}+\text{Δ},E_{0}+\text{Δ})-I2_{Re}^{Tauc}(E,E_{0}+\text{Δ},E_{0}+3\text{Δ}/4)]\} \end{array}\;(27)$$

where:


$$\begin{array}{l} IC_{Re}^{Tauc}(E,E_{r})=\frac{1}{\pi D}\int \frac{[(E_{r}-E)/D]\text{d}E_{r}}{E_{r}^{2}[1+{\left[(E_{r}-E)/D\right]}^{2}]}=\\ \;\frac{1}{2\pi {\left(D^{2}+E^{2}\right)}^{2}}(-4DE\arctan\left(\frac{E-E_{r}}{D}\right)+\frac{2E(D^{2}+E^{2})}{E_{r}}+(E^{2}-D^{2})\ln\left(\frac{D^{2}+{\left(E-E_{r}\right)}^{2}}{E_{r}^{2}}\right)\;\} \end{array}\;(28)$$

$$\begin{array}{l} I2_{Re}^{Tauc}(E,E_{0},E_{r})=\frac{1}{\pi D}\int \frac{{\left(E_{r}-E_{0}\right)}^{2}[(E_{r}-E)/D]\text{d}E_{r}}{E_{r}^{2}[1+{\left[(E_{r}-E)/D\right]}^{2}]}=\frac{1}{2\pi {\left(D^{2}+E^{2}\right)}^{2}}\{4DE_{0}(D^{2}+E^{2}-EE_{0})\arctan\left(\frac{E-E_{r}}{D}\right)\\ \;+[D^{4}+E^{2}{\left(E-E_{0}\right)}^{2}+D^{2}(2E^{2}-2EE_{0}-E_{0}^{2})]\;\ln(D^{2}+{\left(E-E_{r}\right)}^{2})\\ \;+2E_{0}\left[\frac{E(D^{2}+E^{2})E_{0}}{E_{r}}+(E^{2}(2E-E_{0})+D^{2}(2E-E_{0}))\ln(E_{r})\right]\} \end{array}\;(29)$$

$$\;\begin{array}{l} \chi_{2-ind-Tauc}(E)=C\{2IC_{Im}^{Tauc}(E,E_{0}+3\text{Δ}/4)-2IC_{Im}^{Tauc}(E,E_{0}+\text{Δ}/4)+\\ \;16/{\text{Δ}}^{2}[+I2_{Im}^{Tauc}(E,E_{0},E_{0}+\text{Δ}/4)-I2_{Im}^{Tauc}(E,E_{0},E_{0})\\ \;-I2_{Im}^{Tauc}(E,E_{0}+\text{Δ}/2,E_{0}+3\text{Δ}/4)+I2_{Im}^{Tauc}(E,E_{0}+\text{Δ}/2,E_{0}+\text{Δ}/4)\\ \;+I2_{Im}^{Tauc}(E,E_{0}+\text{Δ},E_{0}+\text{Δ})-I2_{Im}^{Tauc}(E,E_{0}+\text{Δ},E_{0}+3\text{Δ}/4)]\} \end{array}\;(30)$$

where:


$$\begin{array}{l} IC_{Im}^{Tauc}(E,E_{r})=\frac{1}{\pi D}\int \frac{\text{d}E_{r}}{E_{r}^{2}[1+{\left[(E_{r}-E)/D\right]}^{2}]}=\\ \;-\frac{1}{\pi {\left(D^{2}+E^{2}\right)}^{2}}\{(E^{2}-D^{2})\arctan\left(\frac{E-E_{r}}{D}\right)+D\left[\frac{\left(D^{2}+E^{2}\right)}{E_{r}}+E\ln\left(\frac{D^{2}+{\left(E-E_{r}\right)}^{2}}{E_{r}^{2}}\right)\right]\} \end{array}\;(31)$$

$$\begin{array}{l} I2_{Im}^{Tauc}(E,E_{0},E_{r})=\frac{1}{\pi D}\int \frac{{\left(E_{r}-E_{0}\right)}^{2}\;\text{d}E_{r}}{E_{r}^{2}[1+{\left[(E_{r}-E)/D\right]}^{2}]}=\\ \;-\;\frac{1}{\pi {\left(D^{2}+E^{2}\right)}^{2}}\{\left[D^{4}+E^{2}{\left(E-E_{0}\right)}^{2}+D^{2}(2E^{2}-2EE_{0}-E_{0}^{2})\right]\arctan\left(\frac{E-E_{r}}{D}\right)\\ \;+DE_{0}\left[\frac{(D^{2}+E^{2})E_{0}}{E_{r}}+(D^{2}+E^{2}-EE_{0})\ln\left(\frac{E_{r}^{2}}{D^{2}+{\left(E-E_{r}\right)}^{2}}\right)\right]\} \end{array}\;(32)$$

Before starting the optimization with the IbridOne method, the function parameters have to be set to initial values good enough to reasonably model *n*( *λ*). Then, assuming *n*( *λ*), *k*( *λ*) values are calculated to perfectly reproduce the *T*( *λ*) spectrum; from the knowledge of *n*( *λ*) and *k*( *λ*), the reflectance spectrum *R* *_cal_* ( *λ*) and the merit function *MF* = $\sum_{i}{\left(R_{cal}(\lambda_{i})-R_{exp}(\lambda_{i})\right)}^{2}/\text{Δ}R_{i}^{2}$ are calculated. The Levenberg Marquardt non-linear least squares curve fitting (offered by the MINPACK library) is then used to minimize *MF* optimizing some parameters of the coating model (e.g. thickness) and of the analytical functions used to describe *n*( *λ*). Note that when using IbridOne the transmittance spectrum is always perfectly reproduced by construction.

In order to choose the most appropriate analytical functions to be used in IbridOne, at least in the initial phase, it is advisable to launch the tracking method first, to identify the wavelength-behaviour of a physically meaningful solution subset (see use case #1 and #4 as examples). Otherwise, the choice of oscillator functions should be driven by the physical properties of the material under investigation: in this case the oscillator parameters in the Simulation TAB has to be manually adjusted to obtain a first rough reproduction of the experimental curve before launching IbridOne (see use case #5).

In any case, users should never forget that the reliability of the obtained *n,k* solutions strictly depends on the correctness of the experimental measurements used as input data. We recommend the reading of Appendix B of the user manual which reports some rules of good practice to avoid the most common errors causing artefacts in spectrophotometric measurements.

### Prediction of the R and T angular behaviour

Historically, in the first years of its development, kSEMAW was massively used for characterizing architectural glazing: day-lighting and energy consumption evaluation for actual rooms requires knowledge of the luminous and energetic parameters of the fenestration, also at off-normal incidence. The direct experimental measurement of transmittance/reflectance at oblique incidence is very cumbersome: the measurement at a given incidence angle must be separately accomplished for the two light polarizations *s* and *p*; several different angles must be considered for sampling the angular range of interest, typically [0,90] deg for architectural glazing and [0,60] deg for the case of Concentrated Solar Power (CSP) ^23^, as well as in Concentrated Photo-Voltaic (CPV).

A simpler approach is offered by the exhaustive optical characterization of the fenestration based on experimental near-normal spectrophotometric measurements and their analysis with a suitable coating optical model. After that, one can numerically evaluate the off-normal incidence behavior ^24^ of any optical features of the device by means of the tools grouped in the *Simulation* TAB.

Unfortunately, often the multilayer structure of commercial products is undisclosed or it is not perfectly known; therefore further information must be obtained by other techniques ^25– 27^, like neutron spectroscopy and ESCA analysis. Anyway, this reverse-engineering process is very difficult and time-consuming.

On the other hand, when the final purpose is the computing of mean values averaged on a standard spectrum, like the energetic and visible parameters of architectural glazing, these mean values are slightly influenced by the realness of the model: as a matter of fact, even if totally unrealistic, optical models perfectly reproducing the experimental measurements at near-normal incidence allow one to predict off-normal incidence features, averaged on solar or visible spectrum, with good accuracy. By virtue of this fact, the Equivalent Model Algorithm (EMA) ^10^ has been proposed. The interested reader will find a detailed discussion and a list of the most useful equivalent models in 10; here we just wish to note that kSEMAW is fully EMA-ready.

**Figure 2.**
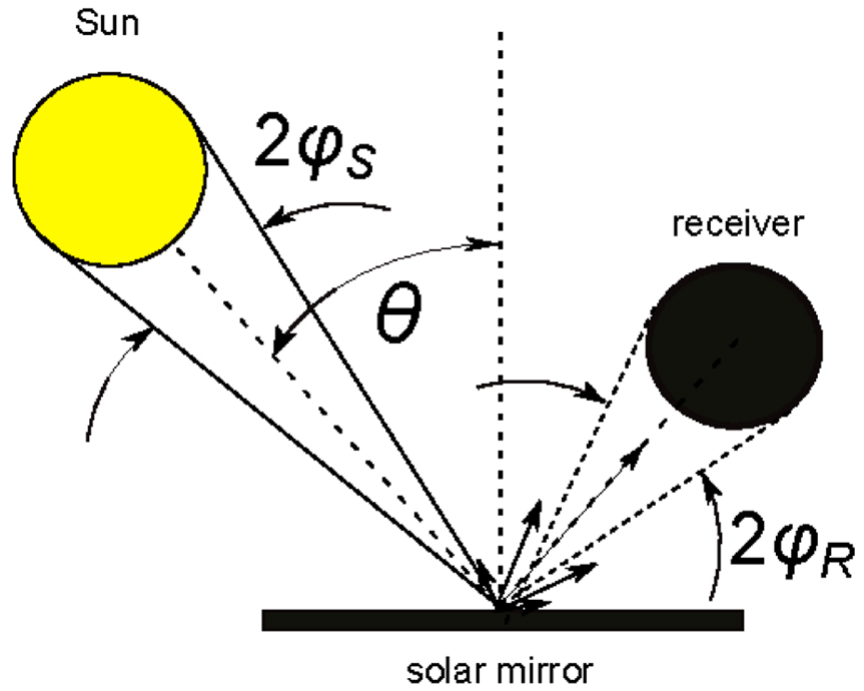
Use case #3: solar mirrors in Concentrated Solar Power (CSP) applications. Θ is the incidence angle; *φ _S_* is the half-angle of solar radiation divergence; *φ _R_* the half acceptance-angle of the receiver.

The situation in CSP and CPV applications is similar but a bit more complex. Mirrors generally work at off-normal incidence to redirect solar radiation on a receiver. As shown in Figure 2 the solar radiation has an intrinsic divergence (the half-angle is about 5 mrad) and the reflected radiation is captured with the acceptance-angle 2 *φ* *_R_* of the receiver (typically *φ* *_R_* ≤ 20 mrad). Therefore the reflectance relevant for CSP/CPV can be referred to as *near-specular*, whose value is generally greater than the genuine *specular* reflectance, strictly related to plane-waves.

An international group of experts is drafting the guidelines for reflectance measurement in SolarPACES Task III ^3^. Even if in the latest Reflectance Guideline ^28^ the recommended procedure consists in measuring only the hemispherical reflectance at near-normal incidence, the importance of also measuring near-specular reflectance is now fully acknowledged, but its experimental measurement is a hard task even for highly specialized optics-laboratories because the divergence of the measuring light beam itself may affect the result ^29^.

Strengthened by the experience gained on glass for building, a more reliable general solution based on a new specific Equivalent Model Algorithm for Solar Mirrors (EMA4SM) was recently presented ^30^: EMA4SM represents the latest evolute adaptation of the Equivalent Model Algorithm (EMA) ^10^ to the specific case of solar mirrors with the target of predicting the angular behaviour versus incidence and acceptance angle of the solar-weighted reflectance.

The algorithm EMA4SM needs a few experimental data measured at near normal incidence, such as:

1. hemispherical reflectance spectrum measured in the solar range (320–2500 nm);
2. near-specular or conic reflectance measured at single wavelength and several different acceptance angles in the range of interest.

Considering the high specialization of both the model and the single wavelength experimental measurements, we have decided to separate this functionality from kSEMAW and arrange the new software SMQexpo for dealing the specific case of solar mirrors. Such software embraces the Open Source philosophy and is freely distributed; interested people can request it by sending an email to marco.montecchi@enea.it.

Therefore actually kSEMAW can be only used for highly specular solar mirrors, i.e. in the cases where near-specular reflectance is equal to the specular one, within the experimental error.

### Implementation and operation

Referring to the user manual for installation details, here we just note that we use to install kSEMAW on PC with Linux operative system (Manjaro or Debian distro). For MS Windows users, dual booting or the installation in a Linux virtual machine are the recommended ways to get kSEMAW working, although in principle the software could be directly installed in Windows and IOS PCs, but we never tried that.

kSEMAW consists of two executables. The first, named ksemaw, C++ written, and based on Qt libraries, manages the graphical user interface (GUI) for data input-output. The second one, named ksemawf, FORTRAN written, runs in background and executes the required computing.

The FORTRAN executable requires the two libraries MINPACK and pgplot.

In order to harmonize the MINPACK sources with the GNU FORTRAN compiler gfortran, a slightly modified version is added to kSEMAW sources; that complies with the MINPACK disclaimer.

The library pgplot is offered as a binary package in most of LINUX distros. As declared in the copyright: “PGPLOT is not public-domain software. However, it is freely available for non-commercial use”, like in the case of kSEMAW.

The C++ executable is devoted to control the Graphical User Interface (GUI), which is based on the open source version of the Qt library, which is offered as binary packages in any LINUX distro.

When launched, two windows pop-up: the GUI and a Linux terminal; the latter shows the progress of the workflow in the two executable C++ ( ksemaw) and FORTRAN ( ksemawf); messages from C++ are preceded by “->”.

**Figure 3.**
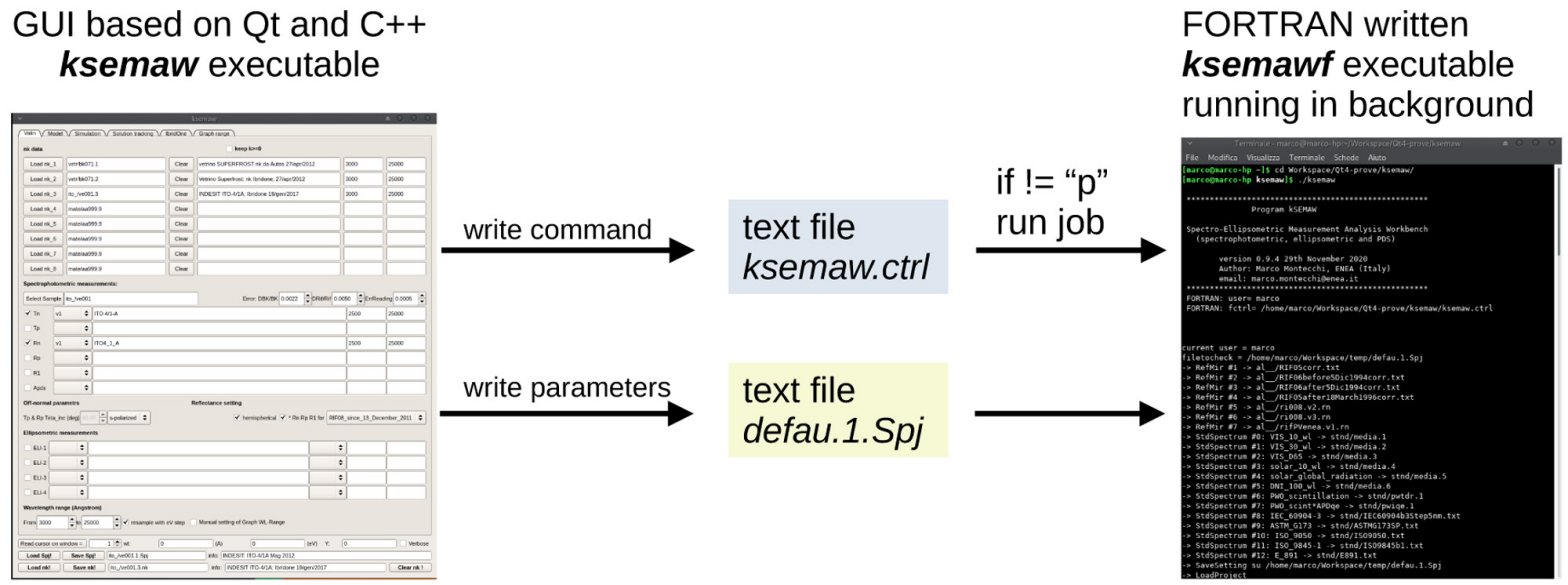
Workflow at the launch of a given command from the kSEMAW graphical user interface.

The two executable interact by means of the two text files ksemaw.ctrl and defau.1.Spj: the former contains the imparted command or the process status, the latter the full parameter set characterizing the current project. Figure 3 describes the workflow at the launch of a given command from the GUI. Once the imparted task has been completed, the FORTRAN executable writes “p” in the file ksemaw.ctrl; the executable C++, alerted for the modification of that file, once found “p”, reloads defau.1.Spj which in the meanwhile has been refreshed by ksemawf. A scheme of the workflow is depicted in Figure 3

**Figure 4.**
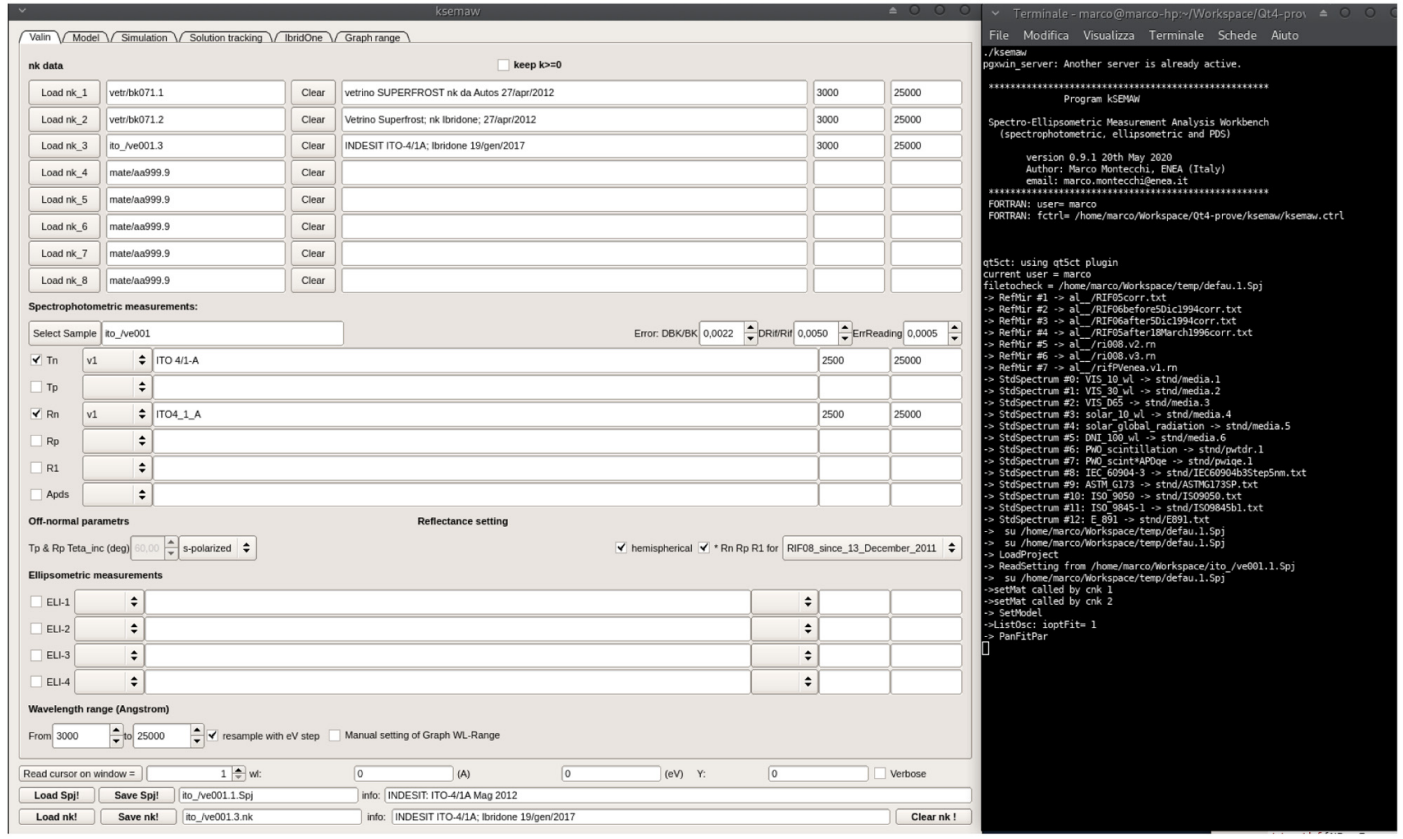
Graphical interface and terminal of kSEMAW.

The GUI, shown in Figure 4, is composed by a top part, structured in 6 “tabs”, ordered in logical sequence, and a bottom part, always visible.

The 6 TABs are:

1. **Valin:** selection of *nk*-files and measurements to be imported in the analysis work bench.
2. **Model:** setting of the optical device model.
3. **Simulation:** simulation of measurements on the basis of known ( *n*, *k*).
4. **Solution tracking:** ( *n _λ_*, *k _λ_*) solution search in tracking mode in the spaces ( *λ*, *n*) and ( *λ*, *k*).
5. **IbridOne:** iterative procedure:
  step-1: *n*( *λ*) model
  step-2: *k*( *λ*) computing from *T*( *λ*) given *n*( *λ*)
  step-3: best-fit of *R*( *λ*) with *n* = *n*( *λ*, *p*_1_, *p*_2_,..., *p* *_M_*) by close loop on these 3 steps.
6. **Graph range:** management of XY plots

The bottom part, always visible, contains

- reading of the cursor position on one of the opened windows
- loading/saving the SEMAW project file ( .Spj)
- loading/saving the file with the solutions *n _λ_*, *k*_λ_ ( .nk)

## Use cases

The complete file-kit of each one of the five use cases presented here are included in the working directories; path and file name are given in each example. Please see *Underlying data*.

### Use case #1: substrate

The evaluation of the complex refractive index of a bare substrate is the simplest case for testing kSEMAW, but at the same time it is a very important task: the most common error in characterizing thin film devices is to neglect the preliminary characterization of the substrate. As a matter of fact, although for a given type of material the real part of the refractive index is fairly constant over different specimens, and well compatible with the values reported in literature, the same cannot be said for the extinction coefficient: in the nearness of absorption tails, such as the UV transmittance cutoff for glass and quartz, the user should not be surprised to find differences much greater than the measurement error, even among substrates belonging to the same batch of supply! Thus if one intends to analyse a series of samples, the preliminary check about the homogeneity of the available substrates and, if necessary, the selection of a group with the same features, are mandatory. In order to properly characterize thin film specimens, one should take care of loading the right *nk*-file of the substrate in the *Valin* TAB, otherwise the found solutions will suffer from more or less evident artefacts.

The use case here presented consists of a sodium calcium glass, 3.9 mm thick, summarized in the kSEMAW project vetr/bk068.1.Spj

As soon as the project is loaded, the experimental transmittance and reflectance spectra related to the specimen here considered, coded as vetr/bk068, are listed in the *Valin* TAB; they were measured with a Perkin Elmer Lambda 900 equipped with a 15 cm integrating sphere in the wavelength range 3000–25000 Å. The reflectance file is not yet normalized to the reference mirror; kSEMAW can do it when “*Rn Rp R1 for” is checked and the proper mirror is selected among the list; please note that the list of reference mirrors can be easily customized following the instruction reported in the user manual.

In general, once *nk*-files and measurements are set, kSEMAW computes the maximum common wavelength range where data of all selected inputs are available. By default, that is the range used for re-sampling both *nk*-file and experimental measurements on a common base of 201 points ^4^, with step in wavelength (Å) or in energy (eV) if the checkbox “resample with eV step” is checked. The latter choice, selected in the current case, is recommended for thin film coatings because it allows to view the interference fringes with an almost regular period.

The model is controlled by the GUI in the *Model* TAB: the actual specimen consists of a single layer, “bulk” type: i.e. e.m. wave propagation is treated by neglecting interference among the several contributions coming from the two interfaces; that is certainly true for the light beam of any UV-VIS-NIR spectrophotometer. The layer thickness is here set to 3.9 mm, that is the experimental value measured with a caliper.

In the current case, by analysing the transmittance spectrum one can note that *T*( *λ*) ≈ 0 for *λ* < 3100 Å ( Figure 6) making it useless for finding solutions; thus it is convenient to resize the wavelength range to 3100–25000 Å.

**Figure 5.**
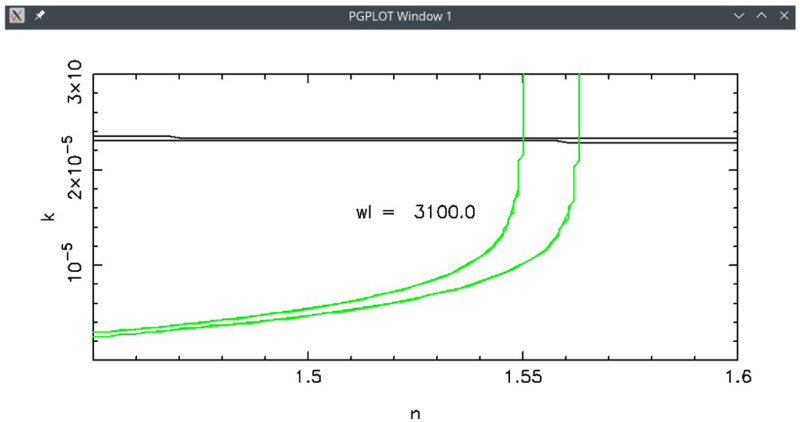
Use case #1: graphical solution search in the complex refractive index ( *n*, *k*) space. The crossing between the two solution-belts of Transmittance (black) and Reflectance (green) represents their common solution at *λ* =3100 Å.

As a general rule it is preferable to start with the tracking-solution method because it does not need the modelling of *n*( *λ*). This method is controlled by the GUI in the *Solution-tracking* TAB. There the user has to set the initial *n*, *k* values to be used for computing; these values can be obtained by searching for solutions in the ( *n*, *k*) space pushing the button “Search in n-k space @”: the considered wavelength is the one specified to the right of this button (the user can set *λ _min_*, *λ* *_max_* or another wavelength in the range). As an example Figure 5 shows the search at *λ* = 3100 Å. In that graph two lines are drawn for each measurement: in this case two (very close) black lines for T and two green lines for R. Each couple of lines limit the belt in which ( *n _λ_*, *k _λ_*) allow to reproduce the experimental values within the error; if the error was null, the belt would collapse to a single line. The intersection area between ( *n _λ_*, *k _λ_*) belts from different measurements represents the common solution area. Here the solution is ( *n* = 1.56, *k* = 2.3 *E* − 05). The same search at *λ* = 25000 Å gives the solution ( *n* = 1.51, *k* = 5.3 *E* − 06).

**Figure 6.**
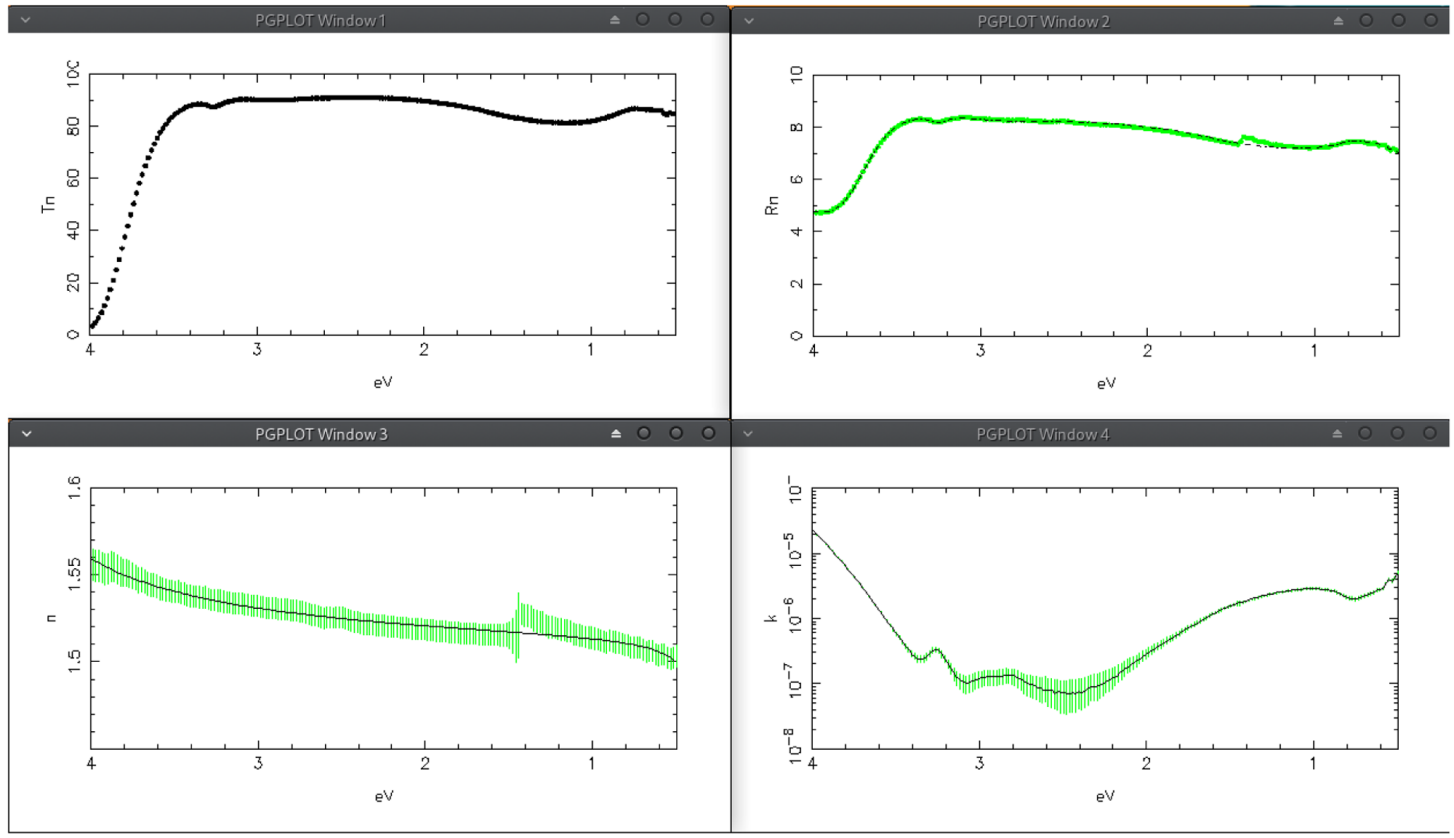
Use case #1: Transmittance (top-left) Reflectance (top-right), refractive index (bottom-left) and extinction coefficient (bottom-right) of sodium calcium glass vetr/bk068. In the bottom row the green vertical bars represent the solutions obtained by the tracking method while the solid lines represent the solutions obtained by the IbridOne method.

The solution-tracking method starts by pressing the button “Search from wlMIN” or “Search from wlMAX”; Figure 6 shows the results: in the first row, drawn as dots, Transmittance and Reflectance spectra; below (as green vertical bars) the computed complex refractive index. The width of the vertical bar is the error due to the errors on the experimental spectra (see the *Valin* TAB in the user manual).

The other computational method, IbridOne, requires the preliminary modelling of *n*( *λ*) with appropriate analytical functions which can be set in the *Simulation* TAB. In the actual case we consider the fit-option composed by: 1) quantum-homogeneous oscillator peaked at 5 eV; 2) Drude term; 3) Flat term. The first term is used to model the growth of *n* towards UV; the second one for modelling its reduction towards IR; the flat term represents the vacuum (see Equation 1) plus the global effect of oscillators with peak energy far from the considered energy range.

Once a suitable fit-option is set, one should surf to the *IbridOne* TAB, which controls the IbridOne computational method. Here one can compose a list of the parameters he wants to consider, and each one of them can be enabled or excluded from the best fit procedure.

The IbridOne procedure runs by pushing the button “Best Fit with IbridOne!”; the new solutions are plotted as the continuous line already depicted in Figure 6. With respect to the solutions obtained by the tracking mode, now the behavior of *n*( *λ*) is totally smooth because it is computed by the analytical functions composing the fit-option; in such a way the reflectance step occurring at about 860 nm, caused by the detector change in the spectrophotometer, has no effect on *n*( *λ*). Conversely the *k*( *λ*) solutions obtained with the two methods are almost identical.

Please note that by definition IbridOne allows the perfect fitting of the transmittance spectrum.

### Use case #2: twin AR coating on glass

The next example was chosen among the several specimens analysed along the optimization of the sol-gel dipping process of porous silica with the aim to obtain solar AR coatings for the glass used to wrap the steel receiver tubes of parabolic trough solar collectors; this process is expected to produce symmetrical twin AR-films on the two sides of the substrate.

The specimen here considered is coded as sipo/ve096; although its features are far from the goal, the case is very useful for learning kSEMAW.

The *nk*-file of the substrate is set at the top of the *Valin* TAB ( vetr/bk048.5); its thickness is 3.3 mm. In the same TAB, experimental Transmittance and Reflectance are set. The common maximum wavelength range is 3000–25000 Å. It is a good practice to start the *nk*-solution search by considering the simplest model. This approach is used in the initial project sipo/ve096.0.Spj and therefore in the *Model* TAB the user will find a model composed by a twin symmetrical single homogeneous film on both sides of the substrate. The film thickness is set to 1875 Å.

The solution-tracking method is controlled by the GUI in the *Solution-tracking* TAB. As already discussed in use case #1, the first step is the evaluation of the ( *n*, *k*) initial values at minimum and maximum of the wavelength range; that is done by pressing “! Search in n-k space @” after the proper selection of wavelength, obtaining ( *n* = 1.48, *k* = 0.008) and ( *n* = 1.38, *k* = 0), respectively for *λ* *_min_* and *λ* *_max_*.

**Figure 7.**
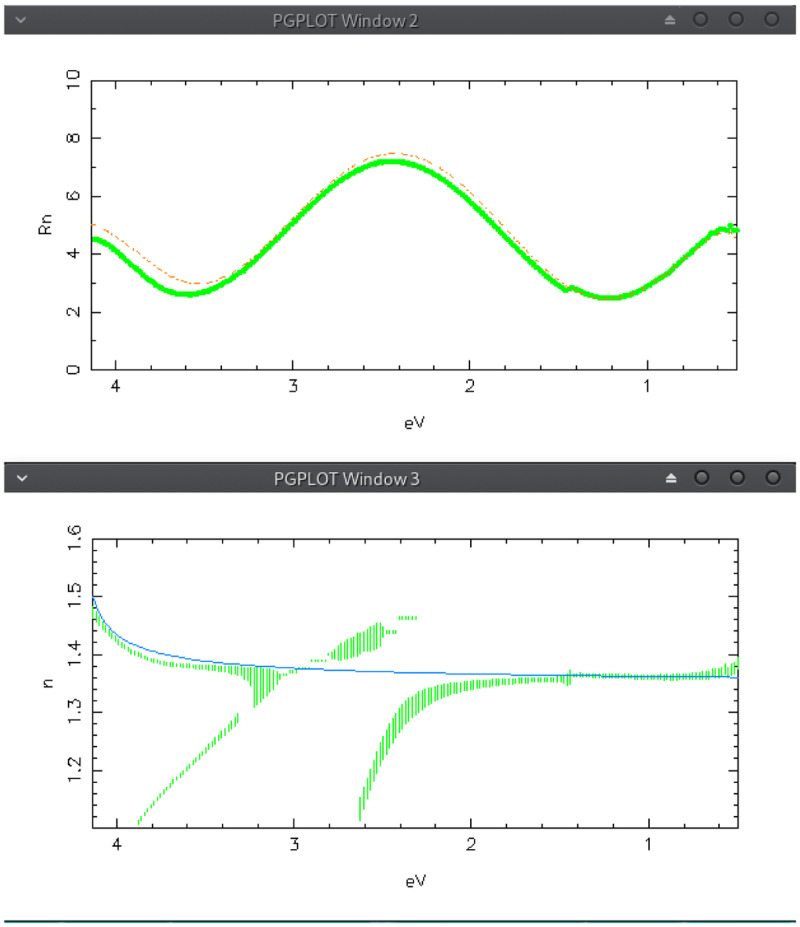
Use case #2: Reflectance (top) and *n*-solutions obtained by the tracking method (bottom) of the AR coating sipo/ve096 by assuming two twin homogeneous films, 1875 Å thick, on the two sides of the substrate. The red dashed line in the top graph is the reflectance computed with the analytical *n*( *λ*) (blue continuous line in the bottom graph) composed by two quantum homogeneous oscillators and one flat term (see text).

Starting from those initial values, the solution-tracking computing is launched by pressing “Search from wlMIN” and “Search from wlMAX”. The results are shown in Figure 7: the *n*-solutions are not connected at the“crossings" related to the extremes *λ* *_m_* = 4 *nd/m* corresponding to *m* = 2 ( *E* ≈ 2.5 eV) and *m* = 3 ( *E* ≈ 2.9); please note that in the case of odd extremes, the related crossings are shifted at lower energy ^12^.

As explained in the papers ^12, 13^, thickness and inhomogeneity of the film can be determined by optimizing the connection of such crossings. To this purpose one should properly modify the film parameters in *Model* TAB, relaunch the solution-tracking in *Solution-tracking* TAB, and observe the effect on the solution crossings related to *m* = 2 and *m* = 3: in the actual case one can easily verify, by trial and error, that the solutions are well connected when thickness and linear *n*-gradient are in the range [1865,1890] Å and [-0.0040,-0.0120], respectively.

After this first important adjustment, the model parameters can be automatically refined by means of the second computing method offered by kSEMAW: the IbridOne algorithm. This method requires the preliminary modelling of *n*( *λ*) with some proper analytical functions, among the several ones offered in the *Simulation* TAB; there one can set the parameters and verify the agreement with the computed *n*-solutions by pushing “Simulate !”. In the current case, the part of the achieved solutions having physical meaning are fairly well fitted with the sum of 3 terms: 1) a quantum-homogeneous oscillator peaked at 5 eV; 2) a quantum-homogeneous oscillator peaked at 4.25 eV; 3) a Flat term. The roughly adjusted function is drawn as a continuous blue line in the bottom graph of Figure 7; the simulated reflectance computed with this function is drawn as a red dashed line in the top graph.

**Figure 8.**
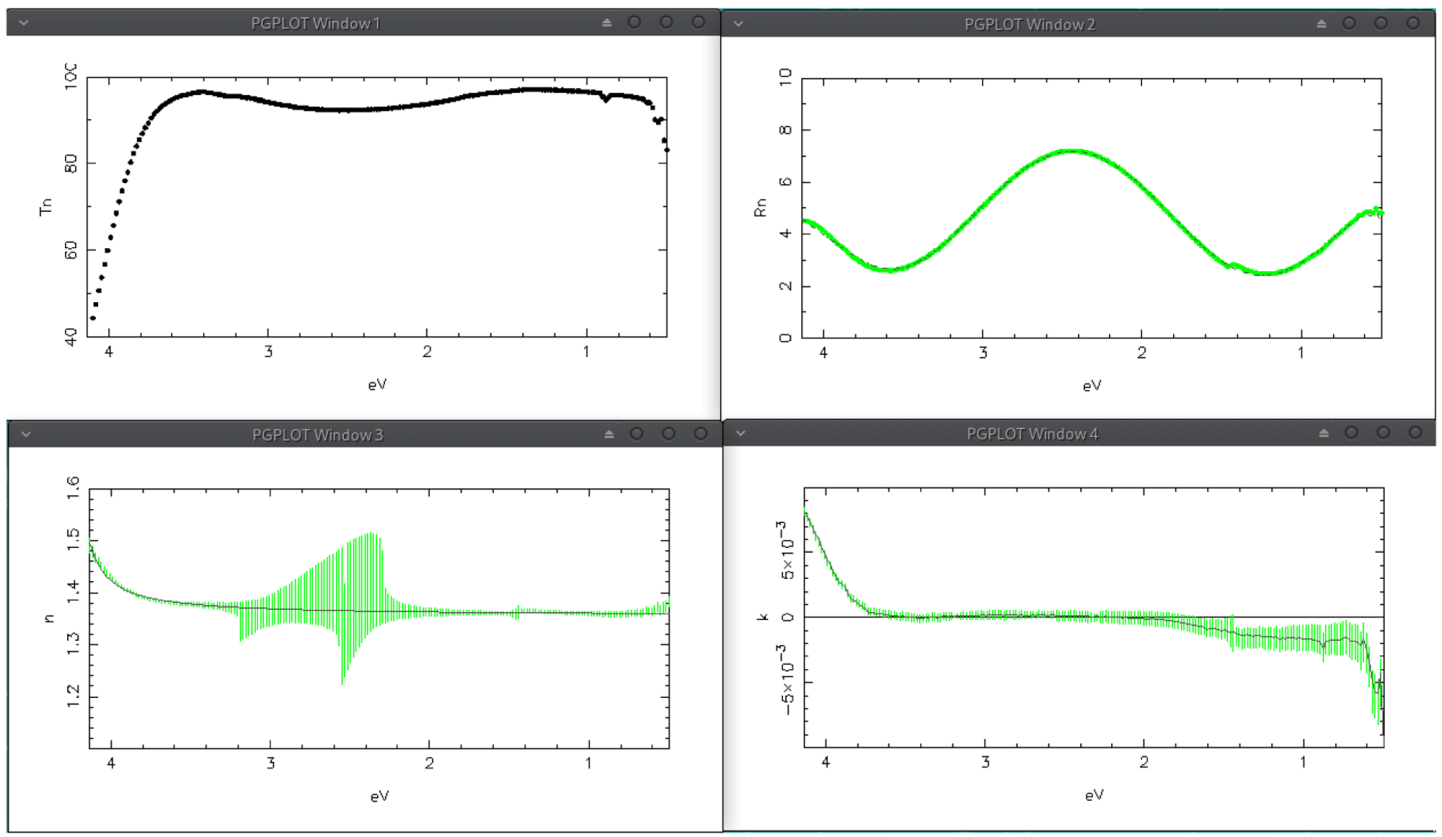
Use case #2: results obtained with the refined optical model (see text). Transmittance (top-left) Reflectance (top-right), refractive index (bottom-left) and extinction coefficient (bottom-right) of twin AR coating on glass.

With these premises, in the “IdridOne” TAB one can set the list of parameters to be viewed and select those he wants to enable for the best-fitting. We found satisfactory results by enabling: thickness, *n*-gradient, and the amplitude *C* parameter of each one of the three terms used to model *n*( *λ*). The final results are saved in the project sipo/ve096.2.Spj and shown in Figure 8. The optimized film thickness is 1876 Å and the *n*-gradient is linear along the film thickness with a negative value (from the substrate to air) of -0.0084 (normalized value; see the user manual). Transmittance and Reflectance perfectly agree with the computed spectra; the continuous lines in *n* and *k* graphs represent the solutions obtained by IbridOne, while the green vertical bars are the solutions computed by the tracking method once the model was optimized by IbridOne; now the *n*-solution crossings are perfectly connected.

Another very interesting point to discuss is the mild negativity of *k* for *λ > *860 *nm* ( *E* < 1.44eV): this artefact is due to the imperfect characterization of the substrate which is just a bit less absorbing then the specimen used for determining the substrate refractive index. As a matter of fact by reducing the substrate thickness to 2.5 mm the artefact almost disappears.

### Use case #3: solar mirror

Here we just consider the simple case of a glass-based highly-specular mirror, coded as ag__/ve010: it consists of a silver layer deposited on a glass substrate 0.95 mm thick; the silver side interfaced with the air is protected with a specific protective treatment ensuring more than 10 years of lifetime in outdoor conditions. Therefore the specimen is specular just only on the glass side, i.e. it is a so-called “second surface mirror”. Since scattering is very low, its features for CSP applications can be simply obtained by the angular behaviour of the solar-weighted specular (back) reflectance, which can be evaluated on the basis of the optical characterization shown in the following.

Let us consider the project ag__/ve010.6.Spj. As usual, the *nk*-file of the substrate is set at the top of the *Valin* TAB ( vetr/bk067.2.nk); the complex refractive index of Silver reported in the literature ^31^ ag__/bk000.9.nk and its customization on the actual sample ag__/ve010.6.nk are also set. The back-reflectance ag__/ve010.v2.r1 is the experimental spectrum used for the characterization.

 In the *Model* TAB the simplest model is already set: a single homogeneous film of silver, thick enough to suppress transmittance (5000 Å), on a glass substrate 0.95 mm thick, with complex refractive index vetr/bk067.2.nk.

**Figure 9.**
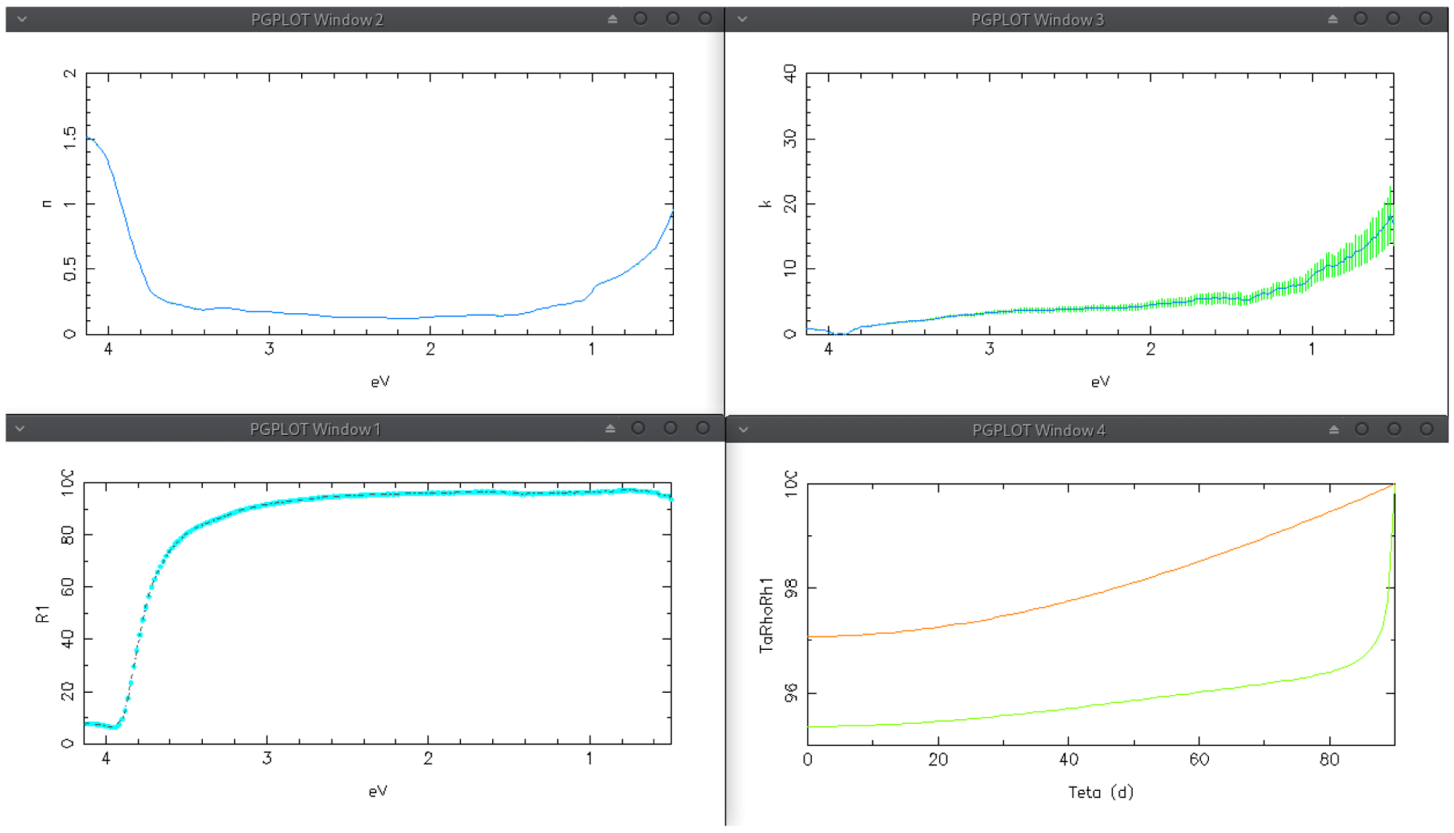
Use case #3: Solarlux mirror ag__/ve010: on top-left, literature values of silver refractive index ^31^; on top-right, extinction coefficient obtained by tracking-solution method. Bottom-left, experimental (dot) and calculated (dashed-line) back-reflectance; bottom-right, solar-weighted (ASTM-G173) front (red) and back (green) reflectance versus incidence angle.

 As a general rule only one of the two unknowns *n* and *k* can be evaluated when just a single experimental spectrum is available; here we evaluate *k*, obtaining values quite close to the starting ones. To that purpose the “strategy” of the tracking mode was set for searching solution from the silver literature values, letting free only *k*, while *n* is kept fixed. The achieved solution are shown in the first row of Figure 9; the calculated *R*1 spectrum (bottom-left) perfectly agree with the experimental one.

 The solar-weighted reflectance versus incidence angle shown at the bottom-right of Figure 9 was computed by setting the “Average type” combo-box (in *Simulation* TAB) to solar spectrum ASTM G173, and pushing the button “Plot <T>, <R>, <R1> vs theta !”; in the graph, reflectance and back-reflectance are plotted in red and green, respectively; the latter is the one of our interest; the average value at normal incidence is displayed in this TAB for both experimental (95.22%) and simulated (95.35%) reflectance; the solar-weighted reflectance increases with the incidence angle.

 The angular behaviour of the solar-weighted reflectance is a very important information for predicting the mirror effectiveness when used to make the concentrators of a specific solar plant. That is a way for comparing the performances of different commercial solar mirrors in a specific application.

### Use case #4: Transparent conductive oxide (IWO)

Transparent conductive oxides (TCO) play an important role in the photovoltaic technology. They are deposited as top layers on heterojunction solar cells and they serve both as antireflective coatings to reduce optical losses and as conductive layers to collect the photogenerated carriers ^32, 33^. Among TCOs, tungsten-doped indium oxide (IWO) is recently attracting much attention ^34^. IWO films are characterized by a wide energy gap >3.5 eV, high transparency in the UV-VIS spectral range and the presence of some absorption in the NIR, related to the density of free electrons in the material.

 The specimen here considered is an IWO sample grown by radio frequency reactive sputtering; the kSEMAW project is iwo/IWO_069.1.Spj.

 The substrate is fused-silica, 1 mm thick; the related *nk*-file is vetr/SLG_sigmaAldrich.1.nk. Transmittance and Reflectance were measured with a Perkin Elemer L950 spectrophotometer equipped with a 15 cm integrating sphere; in this case the reflectance file is already normalized to the reference mirror thus in *Valin* the checkbox “*Rn Rp R1 for” is unchecked.

The common maximum wavelength range is 2500-25000 Å, but the UV limit of the range set in *Valin* must be increased to 2700 Å because at shorter *λ* the transmittance value is zero (within the experimental error) making it useless for evaluating *k*.

 The simplest model consists of a homogeneous film on the substrate. On the basis of the deposition duration and the film growth-rate, the film thickness is expected to be about 93 nm.

Under these assumptions one can launch the solution-tracking method and use the crossing points of the *n*-solutions to improve the model, as already discussed in use case #2. The initial values, obtained by searching solution in the ( *n*, *k*) space, are ( *n* =2.2, *k* =0.5) and ( *n* =2.36, *k* =0.36), respectively for *λ _min_* and *λ _max_*.

 The examined wavelength range includes *m* = 2, 3, 4 crossings: the even ones are well connected, confirming the hypothesis of film homogeneity, while *m* = 3 suffer from thickness underestimation. This crossing point becomes connected by increasing the thickness to about 1010 Å.

 From this point on one can take advantage of the IbridOne method. To that purpose the *n*-solutions having physical meaning must be modelled by analytical functions; a good option is to consider the three terms: Direct-Gap-Cody, Flat, and Drude. The first (third) term models the absorption in UV-blue (NIR) range. 

**Figure 10.**
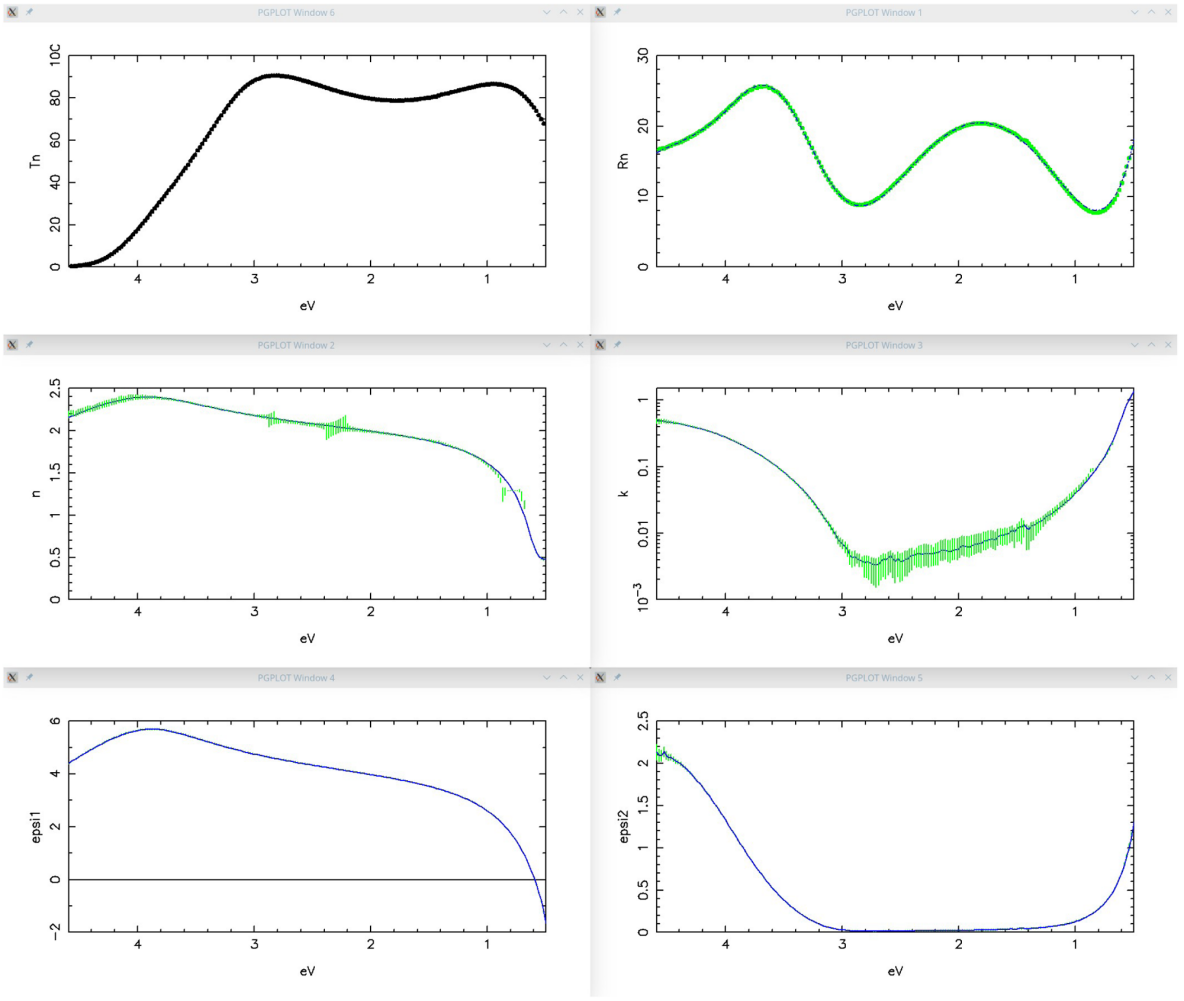
Use case #4: Transmittance (top-left), Reflectance (top-right), refractive index (centre-left), extinction coefficient (centre-right), *ϵ*_1_ (bottom-left) and *ϵ*_2_ (bottom-right) of iwo/IWO_069. The green vertical bars represent the solutions obtained by the tracking method while the solid lines represent the solutions obtained by the IbridOne method.

We run IbridOne several times, gradually refining the parameters, among which the film thickness definitively set to 1014 Å. Figure 10 shows the optimized results: Transmittance and Reflectance experimental spectra are in perfect agreement with the calculated ones on the basis of the complex refractive index shown in the middle row. As usual, the green vertical bars represent the solutions obtained by the tracking method while the solid lines represent the solutions obtained by the IbridOne method.

It has to be noted that kSEMAW gives the possibility to display also the real and imaginary parts of the dielectric constant (see for example the bottom rows in Figure 10) when, in the *Simulation* TAB, the related checkbox is enabled. This is a very useful feature when working on semiconductor materials with extended and structured absorption bands because such a plot can help to configure a suitable oscillators set by comparison with literature data or with a previous fit.

### Use case #5: transition metal dichalcogenide (MoS _2_)

The development of several kinds of new photovoltaic devices would greatly benefit from the availability of a semi-transparent hole transport material (HTM) with a high work function ^35^. MoS _2_ is a good candidate for this role since it has an indirect optical gap at 1.17-1.23 eV ^36^ while the first direct gap is at 1.85 eV ^37^. The indirect gap gives a rather weak absorption so that films with a thickness of a few tens of nanometers are quite transparent for wavelengths greater than 800 nm. MoS _2_ photothreshold (i.e. the energy difference between the vacuum level and the valence band maximum) is quite high being about 5.6 eV ^38^, so that a p-type doped MoS _2_ would show a work function larger than 5 eV, suitable for HTM applications. MoS _2_ was already employed in a monolithic CZTS/Si tandem device where a MoS _2_/FTO/ZnO multilayer structure was used as intermediate contact ^39^ between the top and bottom cells. The MoS _2_ layer used in this multilayer was obtained by metallic Mo sulfurization, as the sample considered in the example below.

The specimen here considered is a film, about 90 nm thick, deposited on a borosilicate Corning glass, 1.1 mm thick with *nk*-file vetr/bk082.1.nk; the specimen is coded as mos2/MoS2_279_BSG. Transmittance and Reflectance spectra were recorded in the UV-VIS-NIR (2500-25000 A) spectral range as described in the use case #4 and, as before, the UV limit of the range set in the *Valin* TAB must be increased to 2900 Å to exclude the data region with almost zero transmittance (unusable to evaluate *k*).

For educational purposes we arranged the initial project mos2/MoS2_279_BSG.paper.9.Spj to show how to set-up, on the basis of experimental and literature data ^36^, a suitable oscillator assortment for modelling the dielectric constant.

As a first reasonable option, based on the known material properties,a set of 5 analytical functions was initially considered in the *Simulation* TAB, including: i) an Indirect-gap and a Direct-Gap-Cody terms at about 1.2 and 1.8 eV respectively, corresponding to the indirect and direct gap energy values, ii) other two Direct-Gap-Cody terms to describe the absorption at higher energies and iii) a constant (Flat) term. From the same TAB, both T and R spectra were calculated and compared with the experimental data: the indirect absorption in the material is so weak that the indirect-gap term was found to have negligible effect in the simulation of optical data and therefore it was removed from the oscillator model.

**Figure 11.**
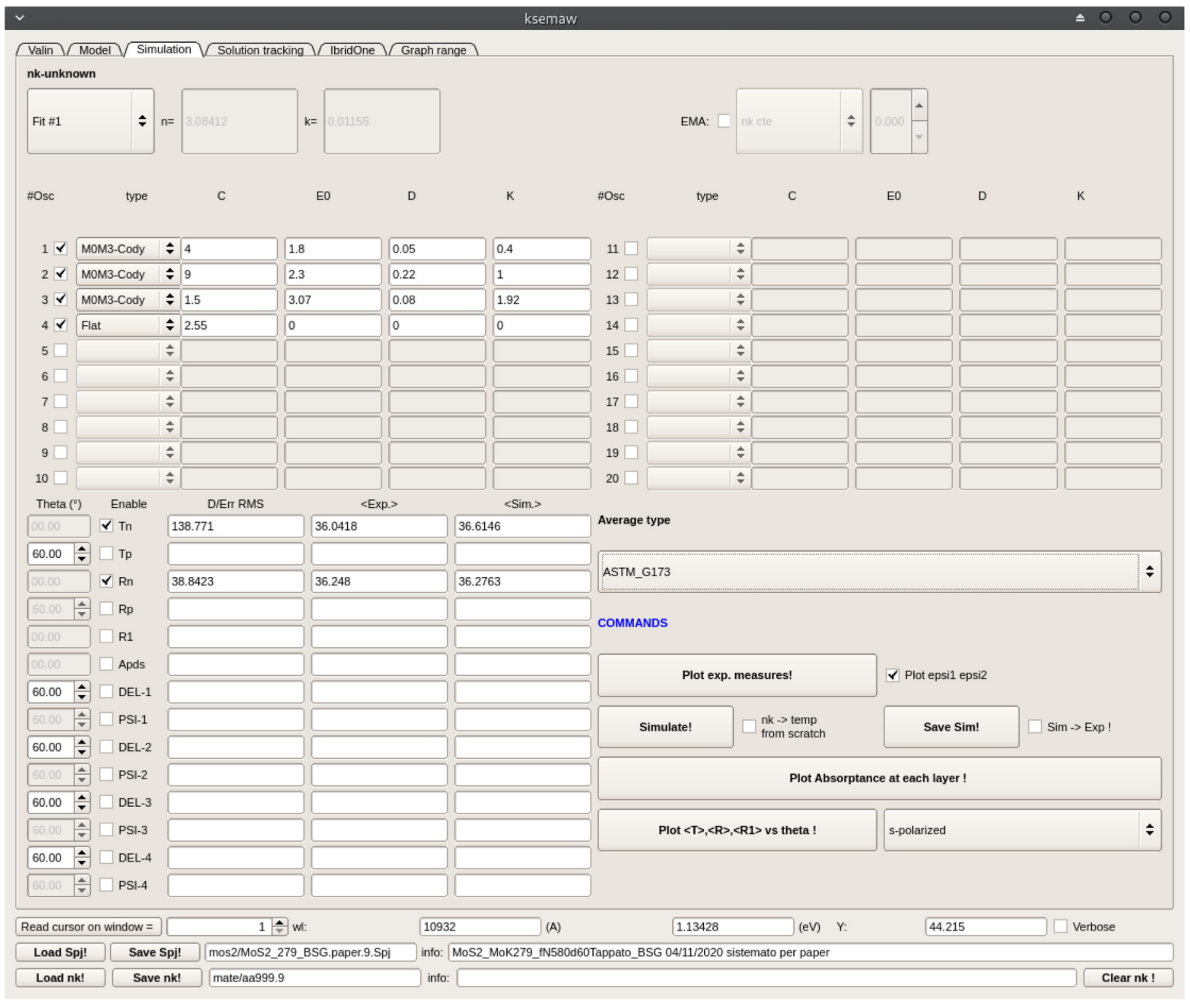
Use case #5: *Simulation* TAB with initial oscillator assortment for modelling the dielectric constant of a MoS _2_ film on the basis of literature data. Parameters *C* (oscillator amplitude) and *K* (the multiplicative factor of the imaginary part of dielectric susceptibility) were roughly manually adjusted for improving the agreement of the computed spectra with experimental Transmittance and Reflectance shown in Figure 12.

**Figure 12.**
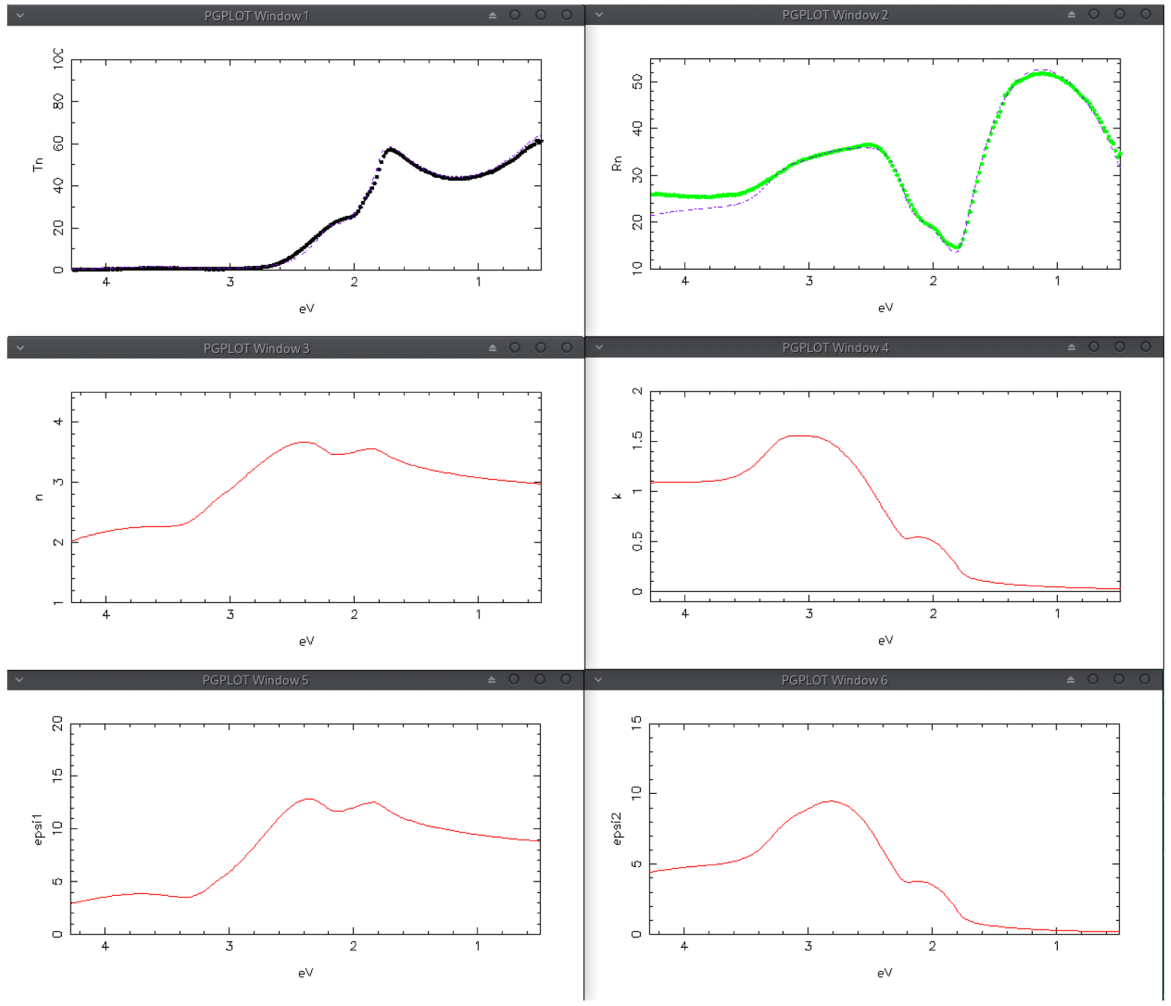
Use case #5, initial situation: Transmittance (top-left), Reflectance (top-right), refractive index (centre-left), extinction coefficient (centre-right), permittivity real part *ϵ*_1_ (bottom-left) and permittivity imaginary part *ϵ*_2_ (bottom-right) of mos2/MoS2_279_BSG. The simulated curves, reported as line, were calculated with the oscillator assortment shown in Figure 11.

**Figure 13.**
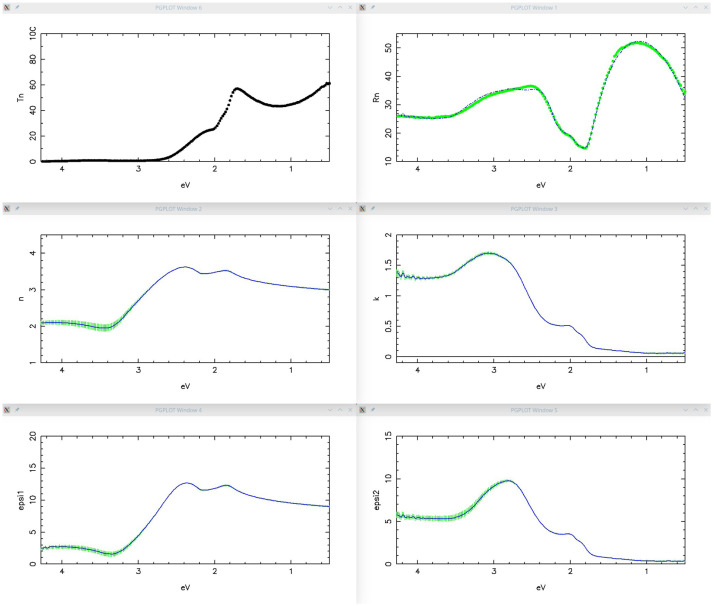
Use case #5, after optimization by the IbridOne method: Transmittance (top-left), Reflectance (top-right), refractive index (centre-left), extinction coefficient (centre-right), *ϵ*_1_ (bottom-left) and *ϵ*_2_ (bottom-right) of mos2/MoS2_279_BSG. The solid lines represent the solutions obtained by the IbridOne method and the green vertical bars their error.

**Figure 14.**
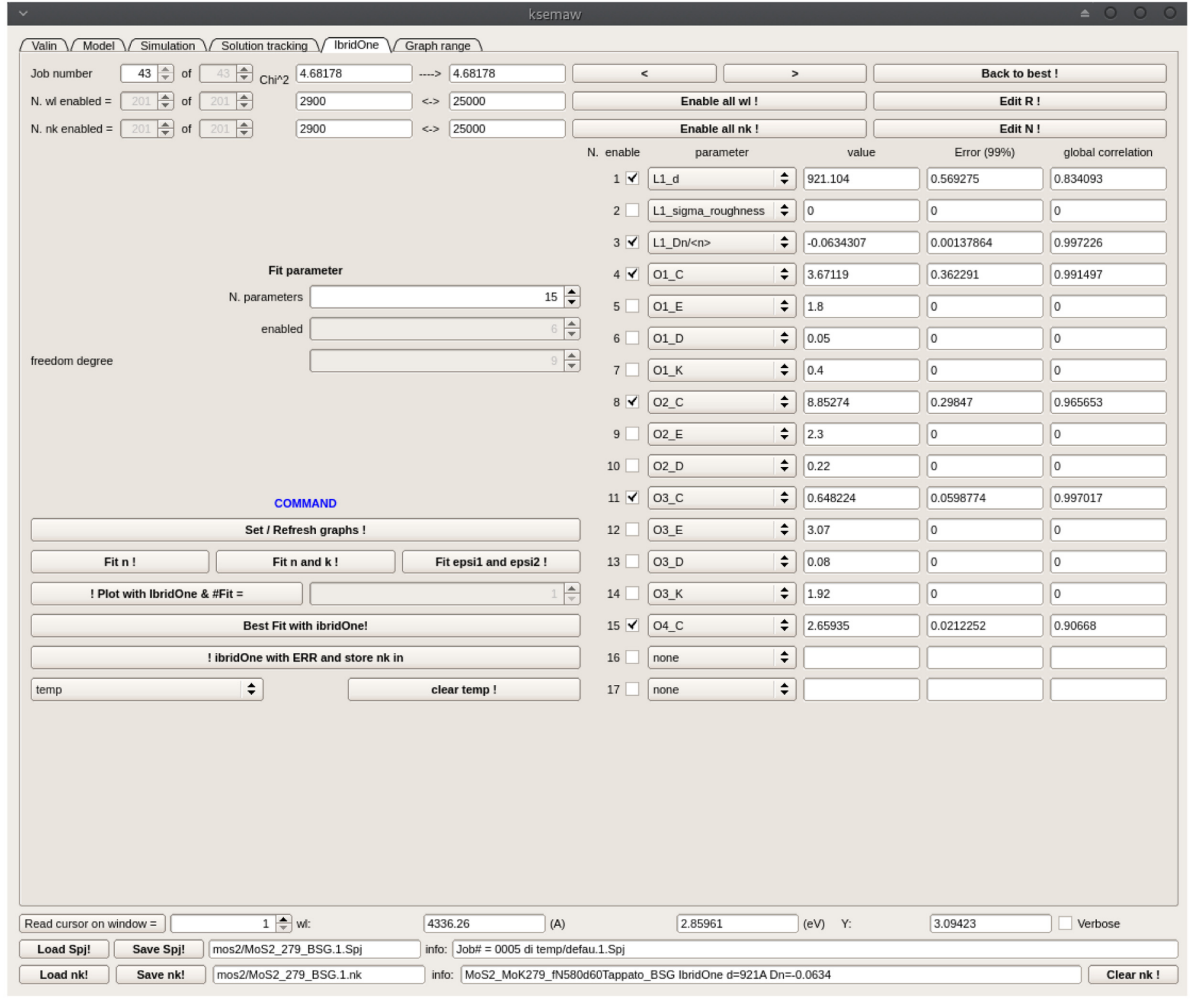
Use case #5: *IbridOne* TAB with the optimized parameters.

After a few manual adjustments, we got the option Fit #1 detailed in Figure 11, giving a fairly good reproduction of all the main features of the experimental spectra, thus validating the proposed oscillator model and its physical meaning. Simulation results are shown in Figure 12. At this point , thickness and *n*-gradient of the film, as well as the *C* parameters of the 4 oscillators composing Fit#1 were further refined by means of the IbridOne method; we found it not useful to enable the others parameters in the best-fitting procedure since a satisfactory agreement between the experimental and fitted curves is obtained. The optimized results are summarized in Figure 13, Figure 14. As previously explained, the calculated transmittance is not shown since the experimental spectrum is always perfectly reproduced by construction using the IbridOne method. The final project was saved as mos2/MoS2_279_BSG.1.Spj.

## Data availability

### Underlying data

Zenodo: mmonty1960/ksemaw: v0.9.6. http://doi.org/10.5281/zenodo.5078212 ^14^. 

This project contains the following underlying data:

- underlying data for use case 1, coded as vetr/bk068
- underlying data for use case 2, coded as sipo/ve096
- underlying data for use case 3, coded as ag__/ve010
- underlying data for use case 4, coded as iwo/IWO_069
- underlying data for use case 5, coded as mos2/MoS2_279_BSG,
- manual_ksemaw-0.9.6.pdf (user manual)

Data are available under the terms of the GNU Affero General Public License version 3. 

## Software availability

Source code available from: https://github.com/mmonty1960/ksemaw

Archived source code at time of publication: http://doi.org/10.5281/zenodo.5078212 ^14^

 License: GNU Affero General Public License version 3

## Notes

^1^kSEMAW adopts the convention of negative sign for the imaginary part of both the complex refractive index and dielectric constant; this convention derives from considering the plane wave solution of the Maxwell equations with positive time expressed as exp[ *i*( *ωt* – **q ⋅ r**)].

^2^According to the Heisenberg uncertainty principle the coherence length is $\Delta l∼\frac{1}{2\pi }\frac{\lambda^{2}}{\Delta \lambda }$ where Δλ is the radiation band-width.

^3^The international network of researchers and industry experts for the development and marketing of concentrating solar thermal power systems and solar chemistry technologies.

^4^That value was set in the early days of software development in ’90, on a workstation HP 9000 equipped with a 100 px wide terminal! Later we continued to keep that value because it does not slow down the solution computing in the selected wavelength range, but it ensures a sufficient sampling-density to represent a spectrum with many interference fringes, provided the step is set to energy.
