# Supplementary material for: KSEMAW: an open source software for the analysis of spectrophotometric, ellipsometric and photothermal deflection spectroscopy measurements
Source: Open Res Eur. 2023 Jan 4;1:95. Originally published 2021 Aug 18. [Version 2] doi: 10.12688/openreseurope.13842.2 (PMC10446058; doi:10.12688/openreseurope.13842.2)
Supplement: Peer review history

# Peer review: Reviewer response for version 1

**DOI:** 10.21956/openreseurope.14925.r27755

**Published:** 2021-11-04

**Authors:** Lars Korte, Alvaro Tejada

**Affiliations:** 1 Young Investigator Group Perovskite Tandem Solar Cells, Helmholtz-Zentrum Berlin für Materialien und Energie GmbH, Berlin, Germany; 2 Departamento de Ciencias, Seccion Fisica, Pontificia Universidad Catolica del Peru, Lima, Peru

The article describes the capabilities of the open source software “kSEMAW”, which is intended for the calculation of optical constants from measurements such as T, R, PDS and ellipsometry. As stated by the authors, the software has been extensively developed over many years and allows for the modelling and analysis of both ideal and non-ideal multi-layer stacks through Fresnel equations and the transfer matrix method. The article is based on the online manual for the software and depicts two different fitting methodologies: a point-by-point approach called the solution tracking method, and a hybrid method named IbridOne. Both are described in detail and are shown in action through various examples. There is also mention of the Equivalent Model Algorithm for obtaining angle-averaged values for T and R.

 The article is scientifically sound and appears quite rigorous in its consideration of the underlying theory. It also demonstrates knowledge of the various experimental concerns and minutia that arise when working with different types of materials, gained through experience.

 The implementation of the solution tracking method is praiseworthy as it highlights the common issues of point-by-point fitting and provides explicit insight on the various (and often diverging) solution branches for n and k. My only question on this matter is whether the film thickness can be fitted using this method, i.e. can the software run through the entire wavelength range, vary the thickness, and try again, in order to minimize the “total” error?

 The situation for the IbridOne algorithm, on the other hand, is a bit confusing. The examples highlight its benefits over the previous method (e.g. reduced sensitivity to instrument artifacts), however it is unclear how this approach is superior (or a suitable alternative) to directly modelling both n and k with oscillator functions, as most commercial software does. The method is quite unconventional in that it uses the dispersion model for only n to extract k from a T measurement, and then uses the R measurement to iteratively validate this. Because it numerically obtains k in this way, some issues become apparent:

1. In comparison to the solution tracking method, k will have the same disadvantages of susceptibility to noise in the measured data and possible unphysical behaviors, without the advantage of being unconstrained by a dispersion model (as the point-to-point models are). This seems like a “worst of both worlds” scenario.
2. Because the fit for T is perfect by definition, it is impossible to assess the quality of the fit with this measurement. Because T is very sensitive to low absorption values (e.g. near the bandgap), as opposed to R, I would say this is a disadvantage, as it makes it less obvious if there are issues with the optical model for the layer stack.
3. Because it is explicitly based on T and R measurements, it is not clear if this methodology could include (or be applied to) additional measurements like ellipsometry or PDS.

In fact, if both n and k were modeled, one would not run into issues such as the negative k in use case #2. Also, it would be possible to fit both n and k in use case #3.

 I’d ask the author to address the previous points, clarify the rationale behind the IbridOne method, and justify its choice over more conventional approaches.

 Finally, a couple of technical questions that could be clarified easily in a revised version of the manuscript:

1. Can the software combine different measurement types in a single fit? For example, if one has R, T and ellipsometry, can it use all of the datasets at once to further constrain the final solution?
2. Regarding the previous point, what happens if the wavelength values of the different datasets overlap but don’t match? For example, one dataset comes from a scanning monochromator with the wavelength values evenly spaced, and another dataset comes from a CCD array with irregular spacing?

Is the rationale for developing the new software tool clearly explained?

Partly

Is the description of the software tool technically sound?

Yes

Are sufficient details of the code, methods and analysis (if applicable) provided to allow replication of the software development and its use by others?

Yes

Is sufficient information provided to allow interpretation of the expected output datasets and any results generated using the tool?

Yes

Reviewer Expertise:

solid state physics; semiconductor physics; physics and technology of thin film deposition and characterization; electronic and optical properties of semiconductors and TCOs; solar cells

We confirm that we have read this submission and believe that we have an appropriate level of expertise to confirm that it is of an acceptable scientific standard.

# Response

**Published:** 2021-11-16

**Authors:** Marco Montecchi

**Affiliations:** ENEA Casaccia Research Center, Italy

Once again we would like to thank the two referees of this second round for their careful review. Our reply to each raised point is following. (Reviewer comments in italics)

*My only question on this matter (the solution tracking method) is whether the film thickness can be fitted using this method, i.e. can the software run through the entire wavelength range, vary the thickness, and try again, in order to minimize the “total” error?*

**Author Reply: **As discussed in User Case #2, the solution tracking method allows the user to optimize the film thickness by optimizing the solution-connection of the odd crossings. Please note that it is up to the user to manually adjust the thickness-value for improving the connections, by trial and error, following the indication reported in the papers [12,13] ; i.e. here the optimization is not automated. The accuracy of such thickness evaluation can be inferred by the thickness range for which the crossing is well connected.

*1. (For the IbridOne method) In comparison to the solution tracking method, k will have the same disadvantages of susceptibility to noise in the measured data and possible unphysical behaviors, without the advantage of being unconstrained by a dispersion model (as the point-to-point models are). This seems like a “worst of both worlds” scenario.*

**Author Reply: **The IbridOne method was introduced to obtain *k*(λ) directly from the experimental data without a detailed model of all the physical mechanisms (excitons, defects, band tails etc) which can be involved in its behavior and which can produce a very articulated spectrum. In general instead *n*(λ) shows a more regular behavior and therefore its modeling is less critical. In our opinion therefore IbridOne is not a “worst of both cases” but a different way to analyze the data: it is automated (while the tracking method is not) and it requires less assumptions than a method where both *n*(λ) and *k*(λ) are modeled and simultaneously fitted. In any case, we are working to add in kSEMAW the possibility to perform this latter (more conventional) fit-option. 

*2. (For the IbridOne method) Because the fit for T is perfect by definition, it is impossible to assess the quality of the fit with this measurement. Because T is very sensitive to low absorption values (e.g. near the bandgap), as opposed to R, I would say this is a disadvantage, as it makes it less obvious if there are issues with the optical model for the layer stack.*  

**Author Reply: **The referee objection is correct if the goal of the analysis is to obtain a detailed physical model of the optical properties of the investigated material. On the other hand the main goal of the IbridOne method is to obtain numerical values of *n*(λ) and *k*(λ) useful for the applications of the material. As for the assessment of the fit quality we point out that the IbridOne method returns also the errors on *n*(λ) and *k*(λ) calculated on the basis of the experimental errors on T and R.

*3. (For the IbridOne method) Because it is explicitly based on T and R measurements, it is not clear if this methodology could include (or be applied to) additional measurements like ellipsometry or PDS.*

**Author Reply: **Currently the IbridOne method can use only the spectrophotometric measurements for T and R (also measured at oblique incidence, polarized or not and from both sample sides). A future implementation of the method will be the possibility to replace R with the ellipsometric angle Psi which in the space (n,k) has a similar behavior and to replace T with the PDS data.

**Reply to the technical questions**

*1. Can the software combine different measurement types in a single fit? For example, if one has R, T and ellipsometry, can it use all of the datasets at once to further constrain the final solution?*  

**Author Reply: **Currently different measurement types can be used together in the tracking method to obtain the (n,k) solution set. As stated above this is not possible for the IbridOne method in the current version but it will be implemented in the next future.

*2. Regarding the previous point, what happens if the wavelength values of the different datasets overlap but don’t match? For example, one dataset comes from a scanning monochromator with the wavelength values evenly spaced, and another dataset comes from a CCD array with irregular spacing?*  

**Author Reply: **Different wavelength spacing in the different datasets is not a problem since the first operation performed by kSEMAW is the resampling of the input data on a common wavelength set.

# Peer review: Reviewer response for version 1

**DOI:** 10.21956/openreseurope.14925.r27647

**Published:** 2021-09-21

**Authors:** Simone Zanotto

**Affiliations:** 1 CNR - Istituto di Nanoscienze, Pisa, Italy

The article "KSEMAW: an open source software for the analysis of spectrophotometric, ellipsometric and photothermal deflection spectroscopy measurements", by M. Montecchi, A. Mittiga, C. Malerba, and F. Menchini illustrates the features of a computer program originally developed for in-house use, and later extended to be exploitable by a larger public including academic and industrial researchers in the fields of optical coating.

 The article recalls the contents of the software user manual, available in the GitHub repository, adding several cases of study, and information about the Equivalent Model Algorithm (EMA) [page 12], that however seems quite off-topic with respect to the main thread of the article.

 Concerning the contents, the article looks complete and scientifically sound, describing the theoretical methods and the numerical implementation. Software installation procedures are better explained in the online manual. The manuscript also provides lengthy analytical expressions for the permittivities; I have checked the consistency between the formulas reported in the article and those reported in the source code only in few cases.

 A few points of the article resulted however slightly unclear to me:

 1. How can the software user analyze the data from a photothermal deflection experiment?

 2. Is it possible to fit simultaneously the n&k data of multiple layers of the coating under analysis? More in general, is it possible to define as fitting parameters a given subset of all the parameters describing the multilayer (e.g., the thickness of a certain layer, and the Lorentz oscillator frequencies of another layer)?

 3. What are the "M_i_cal(n,k)" and similar quantities in the formula for the merit function (MF) [page 5]?

 Finally, could the authors highlight the rationale behind the proposed implementation, possibly commenting on the differences/similarities between the proposed code/method, and that published under the name of "RegressPro", available as open-source code at https://github.com/franko/regress-pro?

Is the rationale for developing the new software tool clearly explained?

Partly

Is the description of the software tool technically sound?

Yes

Are sufficient details of the code, methods and analysis (if applicable) provided to allow replication of the software development and its use by others?

Yes

Is sufficient information provided to allow interpretation of the expected output datasets and any results generated using the tool?

Yes

Reviewer Expertise:

Photonics, metamaterials

I confirm that I have read this submission and believe that I have an appropriate level of expertise to confirm that it is of an acceptable scientific standard.

# Response

**Published:** 2021-09-30

**Authors:** Marco Montecchi

**Affiliations:** ENEA Casaccia Research Center, Italy

First of all we would like to thank the referee for his careful review of the paper. Our reply to the comments follows.

1. *How can the software user analyze the data from a photothermal deflection experiment?*
  The file containing the PDS data can be loaded in the same way as the spectrophotometric and ellipsometric data. After that, the user can simply simulate the PDS data providing an optical model defined by the layer thicknesses and the material refractive indexes. The refractive indexes can be provided as input data files as well as oscillators sets. All the parameters can then be manually optimized to reproduce the experimental data. On the other hand, if the user wants to obtain the refractive index using the solution-tracking or the IbridOne method, the PDS data must be accompanied by another data file as reflectance or Ψ from ellipsometric measurement.
2. * Is it possible to fit simultaneously the n&k data of multiple layers of the coating under analysis? More in general, is it possible to define as fitting parameters a given subset of all the parameters describing the multilayer (e.g., the thickness of a certain layer, and the Lorentz oscillator frequencies of another layer)?*
  In the current version the user can fit the unknown complex refractive index of just one material described by a given set of oscillators. On the other hand, the unknown material can be associated to more than one layer and any other parameter of each layer (thickness, roughness, *n*-gradient, etc) can be defined by the user as a fitting parameters.
3. *Are the "M_i_cal(n,k)" and similar quantities in the formula for the merit function (MF) [page 5]?*
   The merit function MF defined in page 5 refers to a single wavelength value. The index i runs on the considered experimental measurements M_i_exp (typically R and T). M_i_cal(nk) is the value of the M_i measurement calculated using the (n,k) values.

**Rationale:**

  kSEMAW is a new open source software for thin film characterization finally published after about 30 years of development and private use. The software has already been used in various fields of physics, such as thin film optical filters, architectural glazing, detectors for high energy physics, solar energy, and, last but not least, photovoltaic devices. Since the beginning, the software has been conceived to be used by people involved in both growth process and optical characterization of thin film, for which the determination of thickness and complex refractive index of a film is crucial. kSEMAW can load all the most relevant measurements for thin film characterization, such as spectrophotometry ( transmittance and reflectance, at near-normal as well as oblique incidence), ellipsometry and Photo-Thermal-Deflection spectroscopy. kSEMAW manages optical models consisting of up to 9 layers; eventually r oughness, gradient and/or curvature of index profile across the film thickness can be individually set for each one of the 9 layers. Beside the unknown materials, the refractive index of all the other materials can be provided by numerical data files or by setting a suitable ensemble of oscillators. The unknown refractive index can be evaluated in two different ways: 1) numerically, lambda by lambda (solution-tracking method); 2) best fit of reflectance once the extinction coefficient is evaluated by the transmittance (IbridOne method). In the latter method the refractive index can be modeled by a suitable number of oscillators selected among nine different types. The underlined subjects in the rational are not present in the other open-source software Regress Pro.   An important difference between the two softwares concerns the oscillator types available for the fitting procedure. In Regress Pro they are only three: • Cauchy • Lorentz • Tauc-Lorentz We note that the Cauchy oscillator is just an heuristic function and the Tauc-Lorentz oscillator is based on a epsilon_2 proportional to (E-Eg)^2/E^2 which is suitable for amorphous or indirect-gap semiconductors but not for direct gap materials. KSEMAW instead has a richer assortment of oscillators suitable for both direct and indirect gap materials: • Flat • Lorentz • Quant-homo • Quant-inhomo • Drude • Direct Gap Cody • Direct Gap Tauc • Indirect Gap Cody • Indirect Gap Tauc Finally we want to point out that the broadening effect in the Tauc-Lorentz oscillator used by Regress Pro is obtained by a simple multiplication by a Lorentz oscillator centered on the maximum of the joint density of states while in kSEMAW the same effect is obtained by convolution with a single oscillator epsilon_2 which is more physically sensible.
